# Metal–organic framework-stabilized organic anion radicals that electrocatalytically convert CO_2_ into multi-carbon products

**DOI:** 10.1039/d6sc06501e

**Published:** 2026-10-30

**Authors:** Naor Cohen, Rotem Balilty, Aviv Diga, Adi Fryder, Ran Shimoni, Ronit Lavi, Idan Hod

**Affiliations:** a Department of Chemistry and Ilse Katz Institute for Nanoscale Science and Technology, Ben-Gurion University of the Negev (BGU) Beer-Sheva 8410501 Israel hodi@bgu.ac.il; b Department of Chemistry, Faculty of Exact Sciences, Bar-Ilan University Ramat Gan 5290002 Israel

## Abstract

The electrochemical reduction of CO_2_ to multi-carbon (C_2+_) products remains a major challenge due to the high activation barrier associated with C–C bond formation and the limited availability of selective electrocatalytic platforms. Herein, we demonstrate that Metal–Organic Frameworks (MOFs) can stabilize electrochemically generated organic anion radicals that drive heterogeneous CO_2_ conversion through single-electron transfer pathways. Specifically, benzonitrile-functionalized UiO-66 (UiO-66-CN) was employed as a porous scaffold that spatially isolates redox-active organic sites, enabling the heterogeneous electrochemical formation of benzonitrile anion radicals. These MOF-tethered radicals activate CO_2_ to form CO_2_^−^˙ intermediates, which subsequently undergo C–C coupling to yield a C_2_ product, oxalate, with faradaic efficiencies reaching 85%. *Operando* scanning electrochemical microscopy (SECM) directly detected CO_2_^−^˙ during catalysis, while *operando* infrared reflection absorption spectroscopy measurements revealed catalyst–CO_2_ interactions consistent with an inner-sphere electron-transfer mechanism. Beyond oxalate formation, the generated CO_2_^−^˙ species were harnessed for electrocatalytic styrene carboxylation, affording phenylsuccinic acid and 3-phenylpropanoic acid with total faradaic efficiencies exceeding 50%. These findings establish MOF-stabilized organic anion radicals as a versatile metal-free heterogeneous catalytic platform for promoting selective CO_2_ activation and complex carbon–carbon bond-forming reactions, to generate value-added multi-carbon products.

## Abstract 2

MOF-tethered, stabilized organic anion radicals activate CO_2_ to form CO_2_^−^˙ intermediates, which subsequently undergo C–C coupling to yield C_2_ products, such as oxalate and carboxylated styrene.

## Graphical abstract

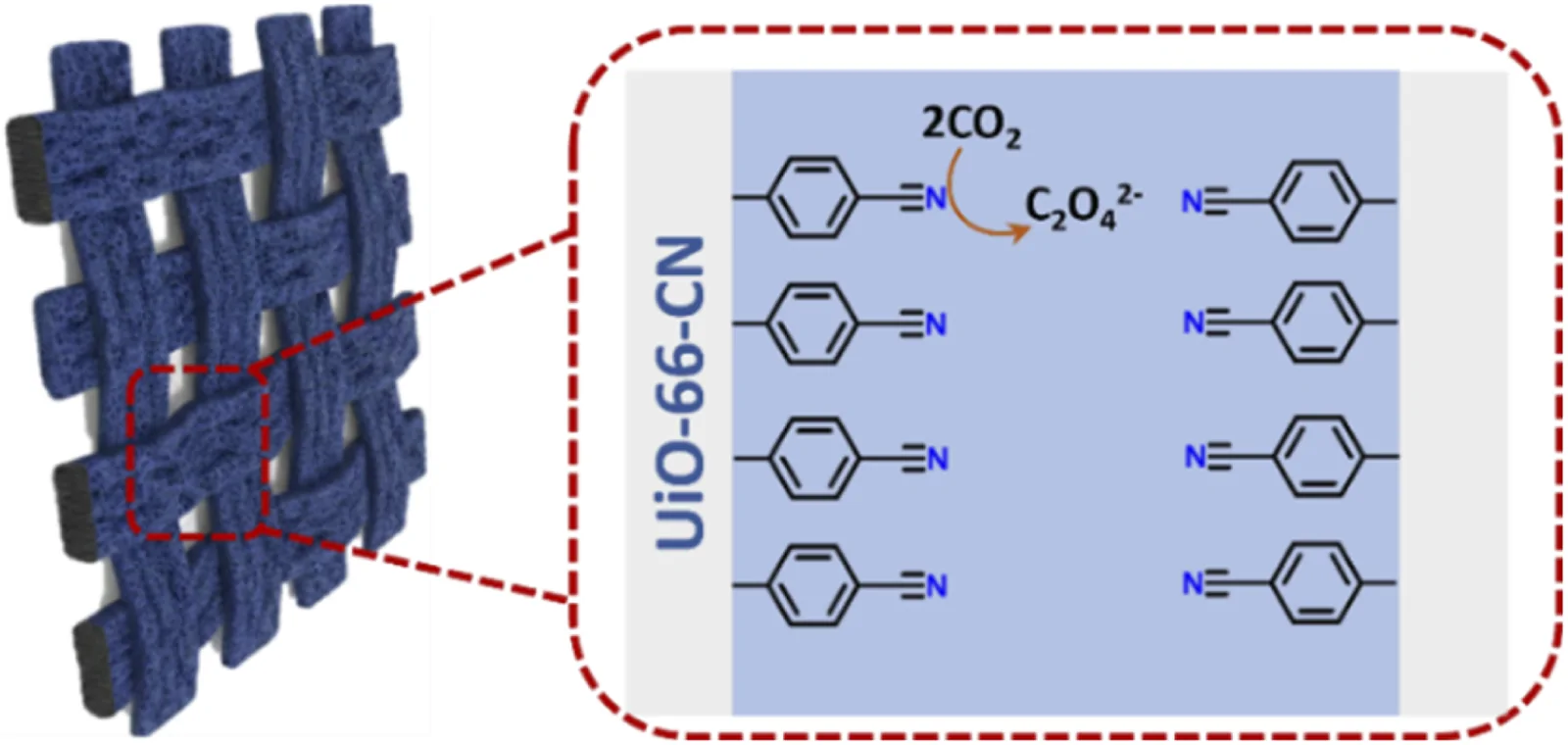

## Introduction

The electrochemical CO_2_ reduction reaction (CO_2_RR) represents a promising strategy for addressing the climate change challenges, by harnessing excess emitted CO_2_ back to the carbon cycle, and its conversion into value-added products.^1–4^ Indeed, in recent years substantial advancement was achieved in the understanding, design, and application of efficient electrocatalysts capable of converting CO_2_ into single-carbon (C_1_) 2e^−^/2H^+^ products, CO,^5–7^ and HCOOH.^8–14^ However, achieving highly active and selective CO_2_RR to multi-carbon (C_2_^+^) products (which are highly desirable due to their superior economic value, increased energy density, and diverse industrial applications) still faces significant challenges. First, CO_2_ reduction to C_2_^+^ products requires the precise coordination of multiple electrons/protons coupled steps, thus creating numerous possible reaction intermediates and competing pathways that can lead to undesired byproducts. Second, the C–C coupling step, essential for forming multi-carbon products, represents a critical kinetic bottleneck with high activation barriers. Indeed, for the production of highly reduced multi-carbon CO_2_RR products, mainly Cu-based electrocatalytic materials are able to show promising activity, albeit with limited selectivity.^15–24^ Hence, to overcome these challenges, there is a need to design new approaches for electrocatalytic schemes capable of generating multi-carbon CO_2_RR products.

In that respect, previous pioneering work by Gennaro and co-authors has demonstrated that anion radicals of unsaturated organic compounds (*e.g.* aromatic nitriles and aryl-benzoates) generated electrochemically in aprotic media, can act as efficient single electron donors.^25^ These organic radical anions serve as homogeneous electrochemical redox catalysts that reduce CO_2_ to a C_2_ product, oxalate. Specifically, application of sufficient cathodic potential generates radical anions having more negative redox potential than the 1-electron activation of CO_2_ to its corresponding anion radical (CO_2_^−^˙). As a result, the as-generated organic radicals will reduce CO_2_ to CO_2_^−^˙, which in turn can dimerize to form oxalate. In other words, this research had suggested that active metal electrocatalysts could be in principle replaced by redox-active organic moieties to achieve high selectivity for C_2_ products. However, practical realization of such homogeneous organic anion radical redox catalysts will necessitate the development of porous platforms which enable their assembly in a heterogeneous fashion while preventing rapid catalyst deactivation through fast radical decay pathways between adjacent catalytic sites. As such, we have postulated that Metal–Organic Framework (MOF)^26^ based scaffolds would be a promising candidate for the immobilization of these organic redox catalysts due to (i) their high porosity,^27,28^ which allows the assembly of large areal concentration of molecular electrocatalysts,^29–41^ and (ii) unlike typical porous materials, the unique structural nature of MOFs imposes intrinsic spatial separation of neighbouring MOF-immobilized catalysts, which may serve to stabilize electrogenerated radicals in aprotic solvents.^42,43^

In this work, a Zr_6_-oxo based MOF, UiO-66,^44,45^ was post-synthetically modified with benzonitrile moieties (termed UiO-66-CN), serving as a model system of organic redox catalysts for anion radical-based single electron transfer CO_2_ activation. Electroreduction of the resulting UiO-66-CN system generated a benzonitrile anion radical (BnCN^−^˙), thus facilitated the formation of a CO_2_ anion radical (CO_2_^−^˙), and in turn allowed efficient production of a CO_2_RR C_2_ product, oxalate, with a faradaic efficiency (FE) of up to 85% at −2.44 V *vs.* SHE (see Fig. 1). Detailed mechanistic analysis was performed by *operando* electrochemical characterization using scanning electrochemical microscopy (SECM) and infrared reflection absorption spectroscopy in ATR (ATR-IRRAS), confirming the activation of CO_2_ and formation of CO_2_^−^˙ *via* the MOF-tethered BnCN^−^˙ during heterogeneous electrocatalytic operation. In addition, we were able to demonstrate that the UiO-66-CN catalyst could be used for more complex C–C coupling reactions, as evidenced by the CO_2_^−^˙-induced carboxylation of styrene to form phenylsuccinic acid (PSA) and 3-phenylpropanoic acid (3PA).

**Fig. 1:**
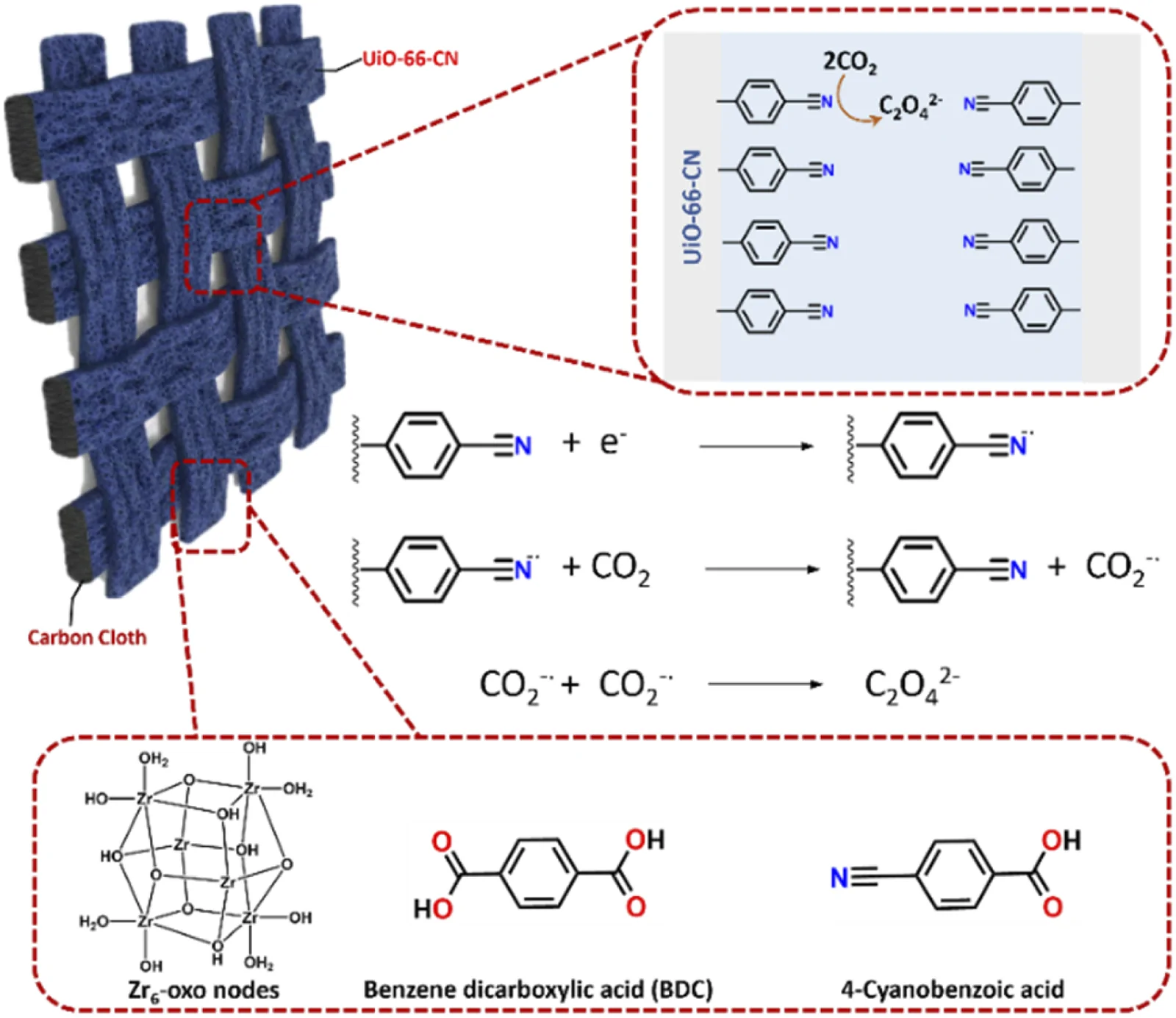
Illustrative scheme of electrocatalytic CO_2_ reduction to oxalate with a UiO-66-CN system, *via* a CO_2_ anion radical dimerization path.

## Results and discussion

The preparation of UiO-66-based thin films was done using a MOF gel precursor, following a previously reported UiO-66 gel synthesis procedure.^46,47^ Briefly, benzene-1,4-dicarboxylic acid (BDC) and ZrOCl_2_·8H_2_O were mixed in a *N*,*N*-dimethyl formamide (DMF) solution containing HCl and acetic acid (CH_3_CO_2_H). The mixture was dissolved by sonication and then placed in an oven kept at 100 °C for 2 hours. Thereafter, DMF was added to the resulting white gel, which was then kept at 120 °C for 24 hours. Next, the obtained UiO-66 gel was washed with ethanol (detailed experimental procedure is found in the SI).^46,47^ The as-formed UiO-66 gel was post-modified with 4-cyanobenzoic acid *via* the solvent assisted ligand incorporation (SALI) method^48,49^ to form the UiO-66-CN electrocatalytic system. In order to quantify the amount of BDC linkers and benzonitrile catalysts per Zr_6_-oxo node in the resulting UiO-66 platform, we combined both inductively coupled plasma optical emission spectrometry (ICP-OES) and proton nuclear magnetic resonance (^1^H-NMR) (for the detailed experimental method and calculations see the SI).^12^ The analysis indicated that the UiO-66 node contains ∼4 missing BDC linkers (corresponding to a BDC : Zr_6_ molar ratio of ∼8). In other words, it means that the formed UiO-66 structure contains up to 4 anchoring spots for post-synthetic ligand incorporation. Indeed, the obtained loading of post-modified benzonitrile ligands was found to be 2.65 per Zr_6_-oxo node (see Fig. S1 and Table S1). The as-synthesized gel was dried and measured by powder X-ray diffraction (PXRD). The diffraction peaks of both samples matched the simulated pattern, confirming the successful synthesis of both UiO-66 and UiO-66-CN gels (Fig. S2). N_2_ physisorption of the UiO-66-CN gel yielded a BET surface area of 718 m^2^ g^−1^ (Fig. S3a). DFT and BJH analysis revealed typical UiO-66 1.2 nm micropores, alongside large 23 nm mesopores, induced by the highly defective nature of the MOF (Fig. S3b and c).

Next, to evaluate the MOF-based catalyst's electrochemical properties, the as-synthesized UiO-66-CN gel was used as a precursor to grow thin films on carbon cloth conductive substrates, using a binder-free drop-casting method (MOF gel volume used for drop-casting was 100 µL). All electrochemical experiments were conducted in a two-compartment H-cell having a conventional three-electrode setup, where the reference (Ag wire quasi-reference electrode) and working electrode (UiO-66-CN) are separated from the counter electrode (Pt foil) *via* a Nafion-117 ion-exchange membrane. The electrolyte solution used was dry DMF (see the SI for additional details) containing 0.1 M TBAPF_6_ supporting electrolyte.

First, as seen in Fig. 2, cyclic voltammetry (CV) measurements under Ar (black curve) and CO_2_ (red curve) atmospheres were performed to examine the electrochemical redox behavior and catalytic potential range of UiO-66-CN. CV of UiO-66-CN in an Ar environment exhibits a quasi-reversible redox peak (at *E*_1/2_ = −2.06 V *vs.* SHE) corresponding to the reduction of MOF-tethered benzonitrile moieties to its corresponding anion radical (BnCN^−^˙). Subsequent potential-step chronoamperometry under Ar revealed that 71.8% of the total BnCN sites are electrochemically addressable (Fig. S4). Upon switching to a CO_2_ atmosphere, the quasi-reversible redox peak is replaced with an irreversible catalytic reduction current, indicating that UiO-66-CN electrocatalytically reduces CO_2_ to form CO_2_^−^˙.

**Fig. 2:**
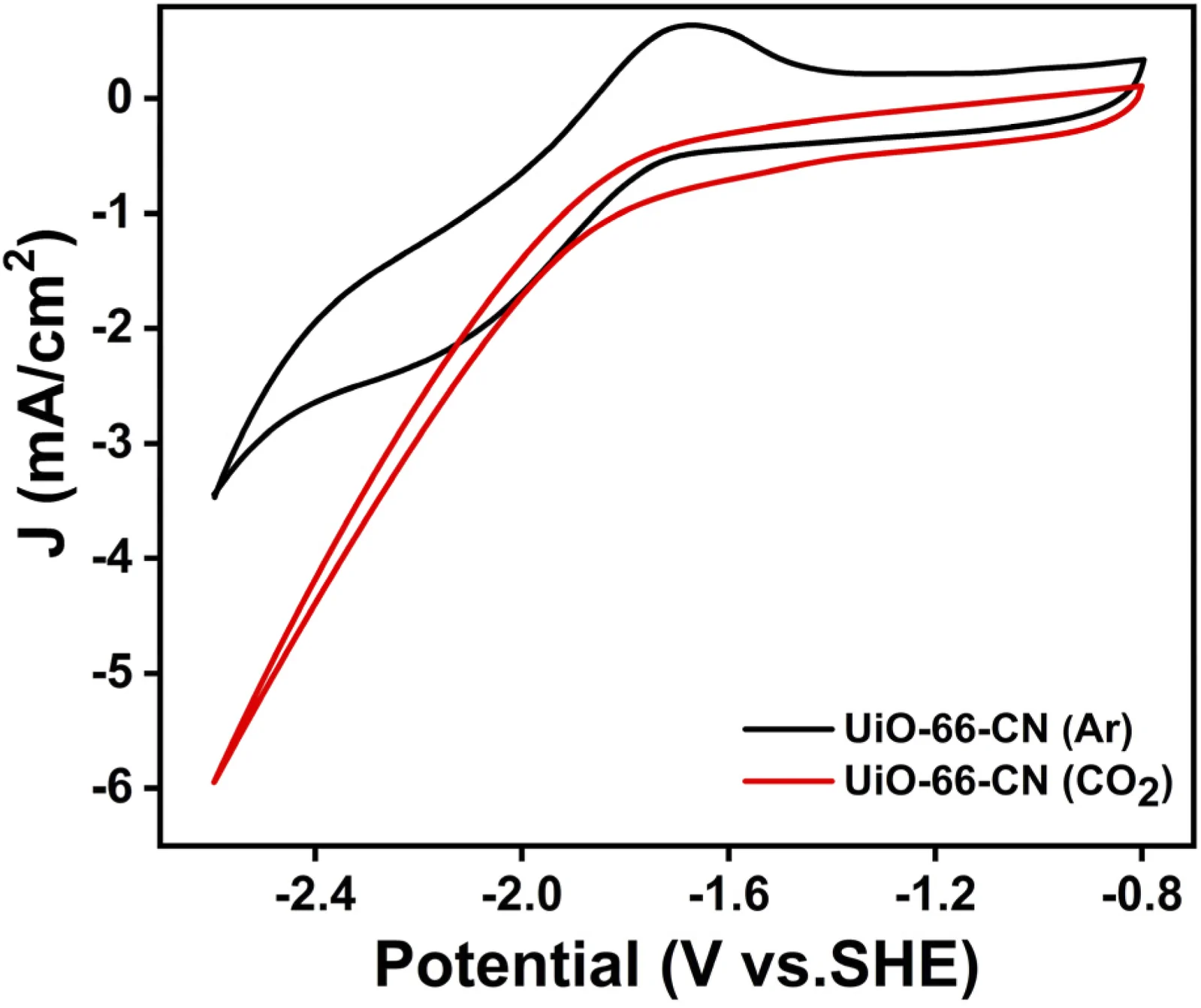
Cyclic voltammograms of the UiO-66-CN electrode, recorded at 500 mV s^−1^ in dry DMF in Ar (black) and CO_2_ (red) environments.

As the postulated CO_2_RR path of our MOF system is the production of oxalate through the dimerization of two CO_2_^−^˙, we were set to evaluate the electrocatalytic activity and selectivity toward oxalate. To do so, chronoamperometric bulk electrolysis measurements were conducted under constant CO_2_ flow (40 mL min^−1^) at applied potentials within the catalytic window, ranging from −1.84 to −2.44 V *vs.* SHE. At each applied potential, a total charge of 3C was passed (Fig. S5 and S6), while the amount of generated oxalate was quantified using high-performance liquid chromatography (HPLC), as shown in Fig. S7 and S8. Fig. 3a and b compares the CO_2_RR performance of UiO-66-CN (red curve) and bare carbon cloth (black curve), plotting oxalate faradaic efficiencies (FEs) and partial current densities as a function of applied potential, respectively. Notably, UiO-66-CN exhibits a high CO_2_RR oxalate selectivity of 85% coupled with a partial current density of −3.6 mA cm^2^ at a potential of −2.44 V *vs.* SHE (coupled with 13% H_2_, as determined using gas chromatography (GC); see Fig. S9 and S10). As opposed to this, bare carbon cloth showed only negligible CO_2_RR activity at the tested potentials (up to 20% FE and significantly diminished partial current densities). Hence, these results clearly demonstrate the essential role of MOF-immobilized benzonitrile redox catalysts in facilitating both the activity and selectivity toward CO_2_-to-oxalate conversion.

**Fig. 3:**
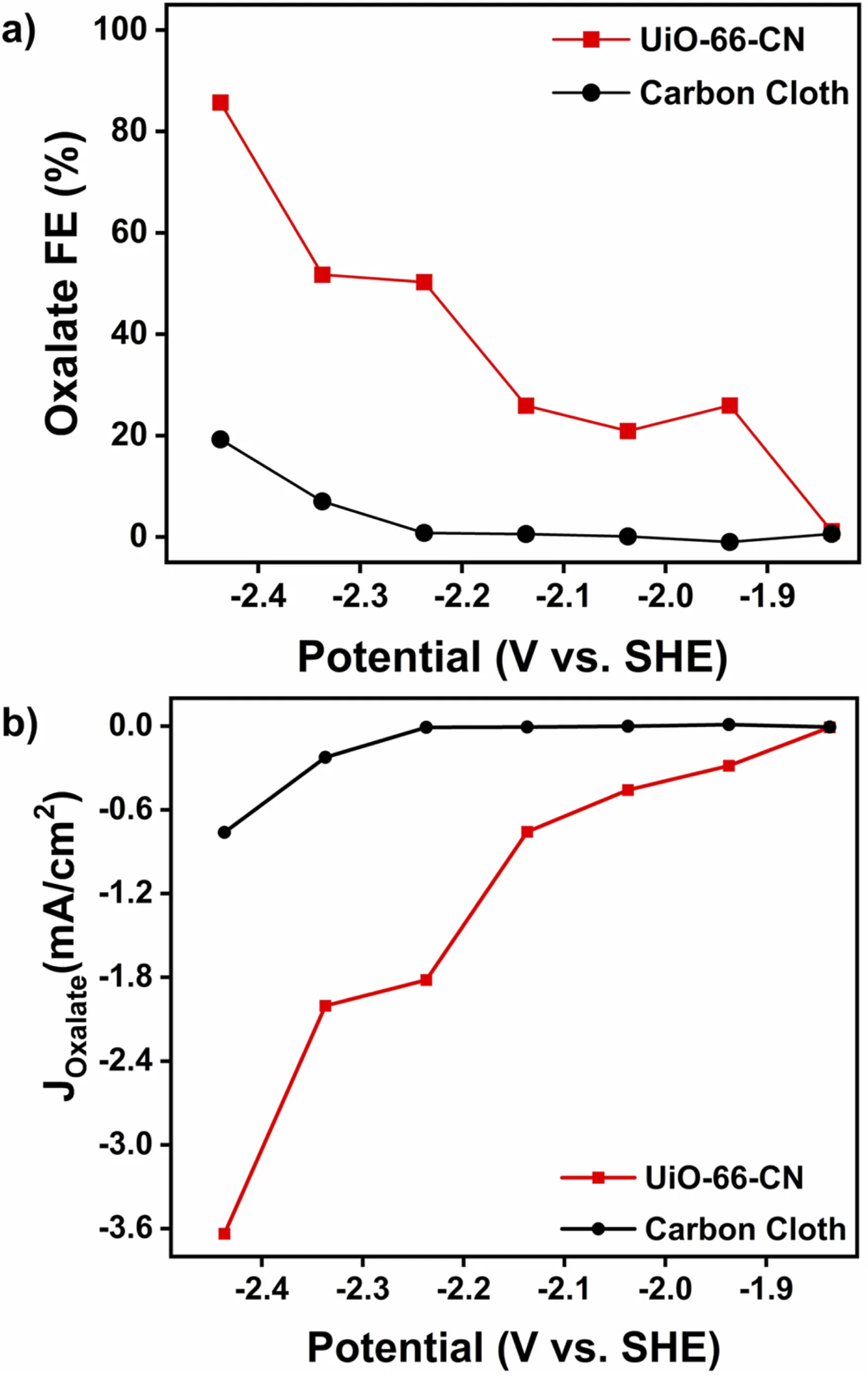
(a) Faradaic efficiency and (b) partial current density for oxalate formation plotted as a function of applied potential, shown for carbon cloth (black) and CC-UiO-66-CN (red).

Upon obtaining these promising electrocatalytic results, we were interested in conducting a more in-depth investigation of the catalytic reaction mechanism. Thus, to confirm the radical-based CO_2_RR pathway to generate oxalate, we sought to achieve direct detection of CO_2_^−^˙, a highly reactive intermediate characterized by an extremely short lifetime and a strong propensity for rapid follow-up reactions, including dimerization or protonation. The transient nature of CO_2_^−^˙ renders its detection inherently challenging using conventional electrochemical methods.^25,50^ To address this limitation, we utilized scanning electrochemical microscopy (SECM),^51^ which enables the localized, real-time interrogation of short-lived species at an ultramicroelectrode (UME) interface.^52^ To do so, as illustrated in Fig. 4a, we have used a SECM setup to conduct an *operando* CO_2_RR-based substrate (UiO-66-CN)-generation tip (10 µm Pt-based UME)-collection characterization. First, an approach-curve experiment was conducted using a solution dissolved fast redox couple (ferrocenemethanol, FcMeOH) to accurately calibrate the distance between the UME tip and the substrate surface. Then, to ensure close proximity between the catalyst and the tip (and thus enable optimal CO_2_^−^˙ collection efficiency) the Pt tip was positioned within the UiO-66-CN MOF layer (see experimental details of approach curves in the SI, and Fig. S11–S14) (substrate-to-tip distance of 5 µm). Thereafter, the UiO-66-CN substrate potential was cathodically scanned in a potential range of −1.44 V to −2.44 V *vs.* SHE to reduce CO_2_ and generate CO_2_^−^˙. Simultaneously, direct CO_2_^−^˙ detection was achieved by holding the tip at a constant potential at −1.14 V *vs.* SHE to effectively oxidize the substrate-generated CO_2_^−^˙ at a mass-transport limited rate. Indeed, as can be seen in Fig. 4b, reductive UiO-66-CN substrate current is followed by oxidative Pt tip current, thus providing a direct confirmation for involvement of the CO_2_^−^˙ intermediate during the electrocatalytic CO_2_RR.

**Fig. 4:**
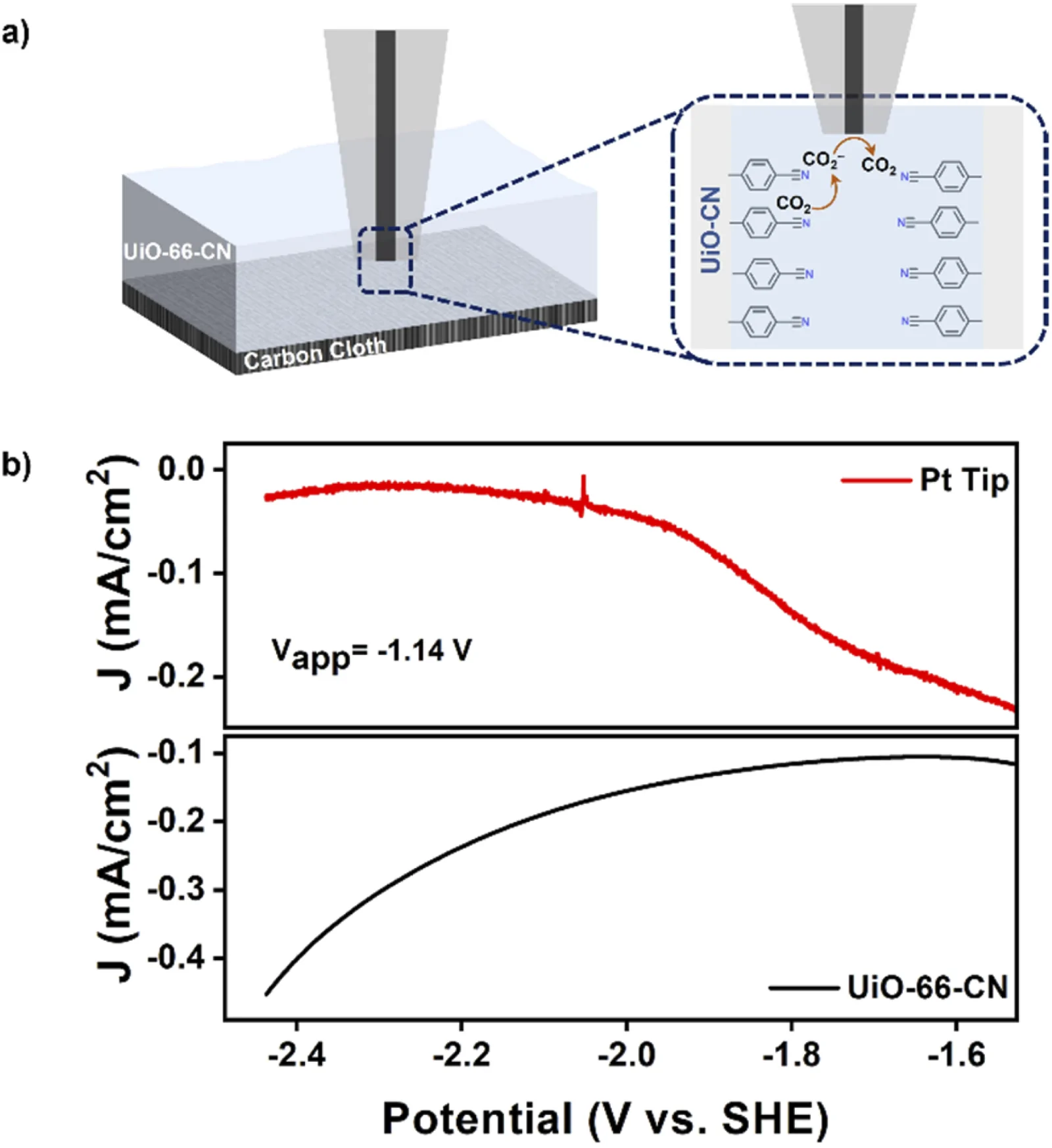
(a) Schematic representation of the *operando* SECM setup for CO_2_^−^˙ detection, demonstrating the positioning of the ultramicroelectrode (UME) tip within the MOF layer; (b) current density at the Pt UME tip held at −1.14 V *vs.* SHE, indicating collected CO_2_^−^˙ (red curve; radius *a* = 5 µm, positioned 5 µm above the substrate), during substrate potential scanning from −1.44 to −2.44 V on UiO-66-CN (black) in dry DMF containing 0.1 M TBAPF_6_.

To substantiate the radical pathway, *ex situ* EPR spin-trapping experiments were conducted on the catholyte using a *N-tert*-butyl-α-phenylnitrone (PBN) radical scavenger (Fig. S15). Cathodic potential under CO_2_ yielded the signature six-line spectrum of trapped CO_2_^−^˙ radicals (*a*N = 15.0 G, *a*H = 5.0 G),^53,54^ while measurements under N_2_ revealed characteristic features of benzonitrile radical anions.^55–57^ These results directly confirm the electrocatalytic generation and involvement of both CO_2_^−^˙ and BnCN^−^˙ intermediates in the catalytic cycle (see SI Fig. S15 for comprehensive spectral analysis).

Next, upon disclosing the involvement of CO_2_^−^˙ as an intermediate during CO_2_-to-oxalate conversion, we were interested in understanding whether UiO-66-CN activates CO_2_ in an inner or outer-sphere manner. To this end, we have also performed *operando* infrared reflection absorption spectroscopy in ATR (ATR-IRRAS) in an Otto configuration, for UiO-66-CN under CO_2_RR operation (see the SI for experimental details). As seen in Fig. S16, at open circuit potential (OCP), two peaks located at 2227 cm^−1^ and 2272 cm^−1^ are observed, corresponding to the C–N stretching of both non-interacted and CO_2_-coordinated MOF-tethered benzonitriles, respectively.^12^ Notably, when applying a cathodic potential of −2.44 V *vs.* SHE (*i.e.* generation of BnCN^−^˙), the 2227 cm^−1^ peak is diminished while the 2272 cm^−1^ peak's intensity is increased. Thus, this indicates that the as-generated BnCN^−^˙ directly interacts with CO_2_ and activates it *via* a single electron transfer (possibly by creating a BnCN-CO_2_^−^˙ adduct). In other words, the *operando* ATR-IRRAS data strongly point to an inner-sphere CO_2_ reduction pathway by the UiO-66-CN MOF electrocatalyst.

To further strengthen the mechanistic understanding, *operando* FTIR measurements were performed under Ar. Upon applying a cathodic potential of −2.44 V *vs.* SHE, a new band emerged at 2117 cm^−1^, assigned to the BnCN radical anion (BnCN^−^˙), accompanied by a simultaneous decrease in the intensity of the parent C≡N stretching band at 2227 cm^−1^ (Fig. S17). These observations provide direct spectroscopic evidence for the electrochemical generation of the benzonitrile radical intermediate.

Building upon our mechanistic understanding, we sought to expand the reaction scope of UiO-66-CN toward more complex C–C coupling reactions. In light of this, we realized that the system capabilities may be extended to the carboxylation of various unsaturated organic molecules *via* the generation and utilization of CO_2_^−^˙. Here, as a proof-of-concept model substrate, we have used styrene. The reaction between styrene and CO_2_^−^˙ could undergo multiple carboxylation pathways, leading to three possible products: 3-phenylpropanoic acid (3PA), 2-phenylpropanoic (2PA) acid and phenylsuccinic acid (PSA), as illustrated in Fig. 5a.^58,59^

**Fig. 5:**
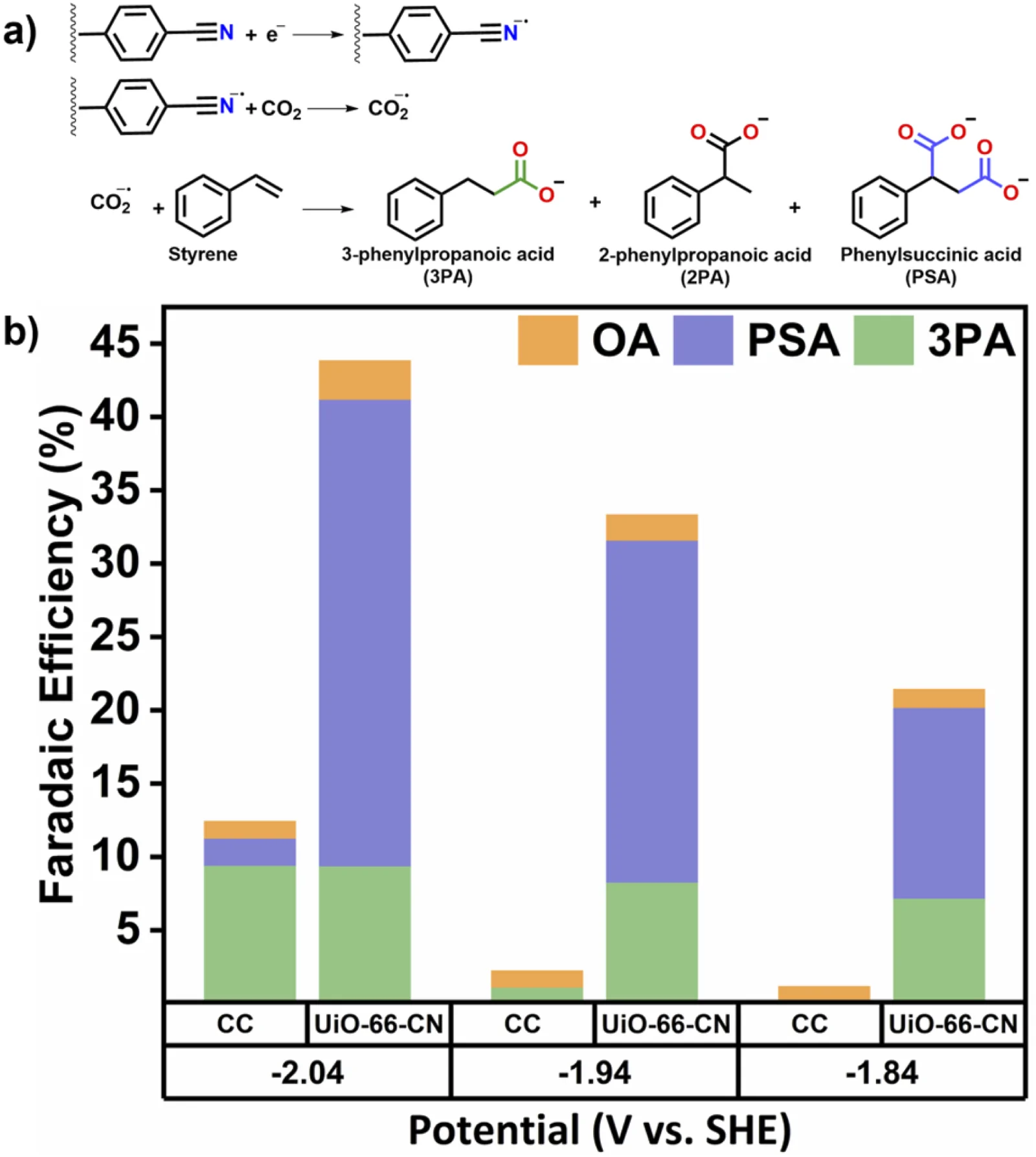
(a) Schematic illustration of electrocatalytic carboxylation of styrene with CO_2_ anion radicals, using UiO-66-CN. (b) Faradaic efficiency for oxalic acid (OA), phenyl succinic acid (PSA), and 3-phenylpropionic acid (3PA) formation as a function of applied potential (comparison of carbon cloth (CC) and UiO-66-CN).

To evaluate the performance of UiO-66-CN (compared to a control bare carbon cloth substrate) toward this C–C coupling reaction, cyclic voltammetry (CV) of UiO-66-CN in the presence of styrene (both with and without CO_2_) was conducted to determine the catalytic potential range while avoiding direct styrene reduction by the MOF catalyst (Fig. S18). According to the measured CVs, a series of chronoamperometric bulk electrolysis measurements were performed in the potential range of −1.84 to −2.04 V *vs.* SHE in dry DMF containing 0.1 M TBAPF_6_ electrolyte solution containing 5 mM styrene (see Fig. S19 and S20). Product quantification, carried out using HPLC (Fig. S21 and S22), showed that for all applied potentials the reaction yielded 3PA and PSA as the main products alongside residual amounts of oxalate (OA), while 2PA was not detected. This product distribution could be explained by the instability of the primary benzyl radical intermediate (which leads to the formation of 2PA) compared to the more stable secondary benzyl radical, which mainly contributes to the formation of 3PA and PSA. As shown in Fig. 5b, a total faradaic efficiency (FE) of up to 44% was achieved for styrene carboxylation at an applied potential of −2.04 V *vs.* SHE (solution of 5 mM styrene and the catalyst's drop-cast volume of 50 µL). PSA emerged as the major product with an FE of up to 30%. Importantly, control experiments utilizing the bare carbon cloth yielded negligible product concentrations across all tested potentials, confirming that the UiO-66-CN catalyst is highly active for driving this targeted styrene carboxylation process.

Next, in order to further optimize the efficiency of the UiO-66-CN system toward electrocatalytic styrene carboxylation, we were set to examine the effect of both styrene concentration and MOF-catalyst loading. First, we evaluated the effect of styrene concentration on the reaction's selectivity. As presented in Fig. S23, at the optimal catalytic potential of −2.04 V *vs.* SHE, a maximum total FE of 46% was achieved at a styrene concentration of 10 mM. Interestingly, one can also notice from Fig. S23, that the 3PA : PSA generation ratio gradually increases as a function of styrene concentration, from 0.2 at 1 mM styrene, up to 0.74 at 20 mM styrene. This concentration-dependent product distribution is consistent with expected reaction dynamics when a radical scavenger (*i.e.* styrene) is introduced. At low styrene concentrations, CO_2_^−^˙ radicals are in relative local excess at the MOF pores, thus favoring the formation of the double-carboxylated product (PSA). In contrast, at high styrene concentrations, CO_2_^−^˙ relative abundance is lowered, suppressing the probability of a second C–C coupling step necessary for producing PSA, and hence enhancing the generation of the singly-carboxylated product, 3PA. Second, we investigated the effect of catalyst loading by varying the volume of drop-cast UiO-66-CN gel on the carbon cloth electrode (between 25 and 100 µL cm^2^). As revealed in Fig. S24, the total product FE exhibited a strong dependence on the catalyst's drop-cast volume. Specifically, an optimal catalyst volume of 75 µL was required to achieve the highest overall FE for the targeted products (52% liquid phase, 7% gas-phase hydrogen *via* GC; Fig. S9 and S25). This optimal loading represents a crucial balance between the surface concentration of MOF-immobilized catalytically active sites (which dictates the rate of initial CO_2_ activation) and the relative mass-transport rates of both catalytic substrates: CO_2_ and styrene (which affects catalytic selectivity). We note that unavoidable side reactions involving styrene self-polymerization processes (as BnCN^−^˙ could also directly activate styrene and initiate polymerization) may account for the lower selectivity of the carboxylation reaction compared to oxalate generation in the absence of styrene.

Finally, we were set to monitor the electrocatalytic stability of the best-performing catalytic UiO-66-CN system (catalyst loading of 75 µL cm^2^ and styrene concentration of 10 mM). To do so, chronoamperometric bulk electrolysis measurement was conducted at −2.04 V *vs.* SHE over a period of 3 hours, during which a total charge of 15C was passed (see chronoamperometric measurement in Fig. S26). Notably, as depicted in Fig. 6, the system exhibited reasonably sustained catalytic activity, with overall FE gradually lowering from 55% to 42% over the course of electrolysis. This sustained activity demonstrates that anchoring the electroactive sites within the MOF scaffold effectively spatially separates them, thereby mitigating deactivation and creating a highly stable platform for radical-driven reactions. Following the 3-hour stability test, to assess the UiO-66-CN electrode's long-term structural and chemical integrity, we characterized it using X-ray diffraction (XRD), scanning electron microscopy (SEM), and SEM coupled with a focused ion beam (SEM-FIB). As shown in Fig. S27, comparison of XRD patterns before and after bulk electrolysis confirmed the preservation of the UiO-66-CN crystallographic structure. In addition, as demonstrated in Fig. S28, SEM and cross-sectional SEM-FIB images showed the retainment of MOF morphology and preservation of UiO-66-CN film thickness (3.6 µm) after the bulk electrolysis stability experiment, respectively. Furthermore, cross-sectional SEM-FIB imaging revealed no apparent changes in the catalyst layer thickness before and after the stability experiment, respectively. Consequently, these characterization studies confirm the high robustness of the UiO-66-CN catalyst under these electrocatalytic operation conditions. To quantify the remaining benzonitrile moieties per Zr_6_-oxo node in UiO-66-CN following the stability test, complementary ICP-OES and ^1^H-NMR analyses were conducted, revealing a mild 11% loss of benzonitrile catalysts, consistent with the observed modest decrease in faradaic efficiency from 55% to 42% (Table S2).

**Fig. 6:**
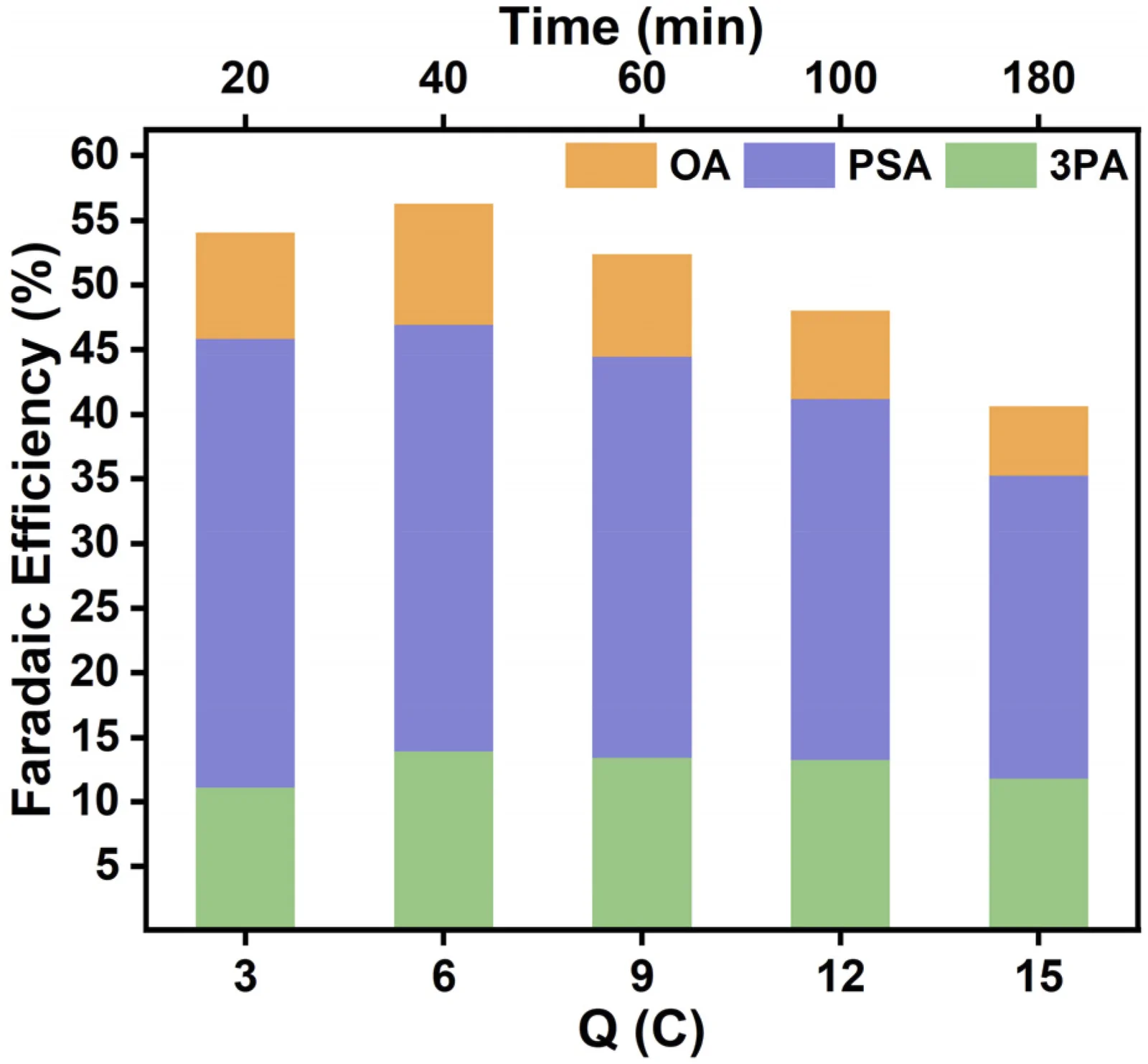
Bulk electrolysis stability test for electrocatalytic styrene carboxylation using UiO-66-CN (at −2.04 V *vs.* SHE), showing the faradaic efficiency (FE) of generated products as a function of time and passed charge.

## Conclusions

Here, we demonstrate the successful utilization of MOF-assembled organic anion radicals for heterogeneous electrocatalytic CO_2_ conversion into multi-carbon products. Specifically, the MOF-based catalyst was constructed from a benzonitrile-modified UiO-66 (UiO-66-CN). Electro-generation of a reactive UiO-66-tethered benzonitrile anion radical (BnCN^−^˙) enabled the electrocatalytic activation of CO_2_ into its anion radical (CO_2_^−^˙), which in turn efficiently dimerized to produce oxalate, a CO_2_RR C_2_ product with a faradaic efficiency (FE) of 85% at −2.44 V *vs.* SHE. Additionally, combined *operando* SECM and FTIR analysis confirmed that the electrocatalytic reaction operates through a radical-based mechanism, in which UiO-66-CN generates CO_2_^−^˙ *via* an inner-sphere CO_2_ activation path. Furthermore, to demonstrate the generality of our UiO-66-CN catalytic platform, we have also expanded its scope to a more intricate C–C coupling reaction, namely electrocatalytic styrene carboxylation. Notably, UiO-66-CN exhibited good selectivity and stability toward the production of phenylpropanoic acid (3PA) and phenylsuccinic acid (PSA), thus highlighting the versatility of the MOF-heterogenized anion radical system. Consequently, this study may pave the way for designing simple, metal-free, heterogeneous electrocatalytic schemes, capable of performing complex multielectron catalytic reactions.

## Author contributions

Naor Cohen, Rotem Balilty, Aviv Diga, Adi Fryder, and Ronit Lavi performed the experiments and analyzed the data. Idan Hod conceived and supervised the project. Naor Cohen, Ran Shimoni, and Idan Hod wrote the manuscript with inputs from all authors.

## Conflicts of interest

There are no conflicts to declare.

## Supplementary Material

SC-OLF-D6SC06501E-s001

## Data Availability

The data supporting this article have been included as part of the supplementary information (SI). Supplementary information is available. See DOI: https://doi.org/10.1039/d6sc06501e.
