# Supplementary material for: Metal–organic framework-stabilized organic anion radicals that electrocatalytically convert CO2 into multi-carbon products
Source: Chem Sci. 2026 Oct 30. Online ahead of print. doi: 10.1039/d6sc06501e (PMC13746452; doi:10.1039/d6sc06501e)
Supplement: Supplementary file 1 [file SC-OLF-D6SC06501E-s001.pdf]

Supplementary Information (SI) for Chemical Science.
This journal is © The Royal Society of Chemistry

Supporting Information

Metal-Organic Framework-Stabilized Organic Anion Radicals That Electrocatalytically Convert CO_2 into Multi-Carbon Products

*Naor Cohen, Rotem Balilty, Aviv Diga, Adi Fryder, Ran Shimoni, Ronit Lavi[2], Idan Hod**

1. Department of Chemistry and Ilse Katz Institute for Nanoscale Science and Technology,

Ben-Gurion University of the Negev, Beer-Sheva, 8410501, Israel.

2. Department of Chemistry, Faculty of Exact Sciences, Bar-Ilan University, Ramat Gan 5290002, Israel.

E-mail: hodi@bgu.ac.il

Experimental Section

Chemicals

Zirconyl dichloride octahydrate ($ZrOCl_2{\cdot}8H_2O$ ≥ 99.5%), terephthalic acid ($C_8H_6O_4$ ≥ 98%) were purchased from Sigma-Aldrich. Glacial acetic acid (AA, CH_4CO_2, ≥ 98%), hydrochloric acid (32 wt%), dimethylformamide (DMF, C_3H_7NO), and ethanol (AR grade) were purchased from Bio-Lab. 4-cyanobenzoic acid (BA-CN, $C_8H_5NO_2$ ≥ 98%) purchased from thermo scientific. Tetra-n-butylammonium hexafluorophosphate ($TBAPF_6$, $C_{16}H_{36}F_6NP$ ≥ 98%) purchased from Angene chemical. 3Å molecular sieves 4-8 mesh purchased from Sigma-Aldrich. CO_2 and Ar gas (≥ 98%) were purchased from Maxima. All electrodes were purchased from FuelCellStore.

Synthetic Procedures

UiO-66 synthesis

In a 50 mL Schott Duran bottle, H_2bdc (2.9 mmol, 0.4820 g, 98%) and $ZrOCl_2{\cdot}8H_2O$ (2 mmol, 0.6440 g, 98%) were dissolved in N,N-dimethylformamide (DMF, 12 mL, 99.8%). Hydrochloric acid (0.3 mL, 32 wt%) and glacial acetic acid (0.4 mL) were subsequently added as modulators to control nucleation and crystal growth. The resulting mixture was sonicated until a clear and homogeneous solution was obtained, ensuring complete dissolution of the precursors. The reaction mixture was then sealed and heated in a conventional oven at 100 °C for 2 h to initiate gel formation. Following this initial step, DMF (12.5 mL) was added to the system, and the resulting gel was thoroughly homogenized using a vortex mixer to ensure uniformity. The mixture was subsequently heated at 120 °C overnight to promote further framework development and cross-linking. In a second dilution step, an additional 25 mL of DMF was introduced, followed by vigorous homogenization. The sample was then maintained at 120 °C overnight to complete gel maturation and enhance structural integrity. The resulting UiO-66 gel was isolated and washed five times with fresh DMF (50 mL per wash) to remove unreacted precursors, excess modulator, and residual solvent trapped within the porous network.[1-3]

Post modification of UiO-66 with 4-cyanobenzoic acid

Post-synthetic modification of the UiO-66 gel was carried out by dissolving 4-cyanobenzoic acid (0.068 mmol, 0.0100 g) in ethanol (2 mL) in a 4 mL glass vial. Subsequently, 2 mL of the UiO-66 gel was added, and the resulting mixture was homogenized. The vial was then sealed and heated at 60 °C for 48 h to enable functionalization of the framework. Following the reaction, the resulting UiO-66-CN material was washed four times with ethanol by centrifugation at 2000 rpm for 5 min per cycle (total wash volume of 4.5 mL per cycle) to remove excess linker and residual impurities. To obtain a concentrated gel suitable for subsequent use, a final centrifugation step was performed at 4000 rpm for 5 min, promoting enhanced sedimentation and removal of excess solvent. After washing, the resulting ink-like gel was redispersed in ethanol using a dilution ratio of 1:2 (gel:ethanol, v/v).[2]

Preparation of dry DMF- for electrochemical measurements

Anhydrous N,N-dimethylformamide (DMF) was prepared by storing DMF (anhydrous, 99.8%) over 20% m/v 3Å molecular sieves (activated by microwave for 7 minutes followed by flame activation under vacuum) for at least 48h under nitrogen atmosphere.[4]

Electrodes preparation

Preparation of carbon cloth electrodes

The electrode (1 cm × 3 cm) was prepared using the drop-cast method by depositing 50 μL of the UiO-66-CN dispersion onto a 1 cm × 1 cm area on one side of the electrode surface. After allowing it to dry for 10 minutes, the electrode was flipped and another 50 μL was deposited on the opposite side. The electrode was then dried overnight to ensure complete solvent evaporation.

Physical Characterization Methods

Powder X-ray diffraction (XRD) of the UiO-66 thin films conducted using PAN analytical's Empyream multi-purpose diffractometer instrument with Cu-K (0.15405 nm) radiation. We measured N_2 physisorption isotherms at 77 K after degassing the UiO-66-CN gel sample for 16 h at 120 °C under vacuum using a Quantachrome Autosorb IQ2 instrument. The surface area of the solid material was determined by utilizing the **Brunauer–Emmett–Teller (BET)** model based on the adsorption branch of the isotherm data points. Additionally, the pore size distribution was obtained by applying the discrete Fourier transform-density functional theory (DFT) at low relative pressure. **Hydrogen Nuclear Magnetic Resonance (H-NMR)** measurements were done on the Bruker DPX-500 instrument. A calibration curve of BDC was prepared using standard samples to evaluate the concentration of BDC and 4-CB in UiO-66 gel and UiO-66-CN, respectively. The different concentration solutions of H_2BDC and UiO-66-CN sample were prepared in 2 M NaOH/D_2O Maleic acid was used as the internal standard. **Inductively Coupled Plasma - Optical Emission Spectroscopy (ICP-OES)** analysis was performed using Spectro ARCOS ICP-OES, FHX22 multi-View plasma (SOP, EOP) instrument to determine the amount of Zr in UiO-66 and UiO-66-CN gel sample. Sample preparation was done by dissolving 1 mg of UiO-66 and UiO-66-CN in 6 mL of concentrated HNO_3 at 120 °C for 12 h. Later, 0.7 mL of the solution was diluted to 9.8mL for ICP-OES measurement. **Scanning Electron Microscopy with Focused Ion Beam (SEM-FIB)** images were recorded using Thermo Scientific Dual-Beam system. The ion beam and the electron intersect at a 52° angle near the sample surface. The samples were first coated with layer of Au (9 nm) and then analyzed by SEM-FIB. During the analysis, the area under investigation was coated first with 150 nm of Pt by electron beam deposition and, on top of that, 1500 nm of Pt by ion beam assisted chemical vapor deposition. Next, a small area was cut by the sputtering of the focused ion beam to visualize the cross-sectional thickness of the membrane. **High-Performance Liquid Chromatography (HPLC):** measurements were conducted using Agilent 1260 Infinity II. The column Hi-Plex H 300 × 6.5 mm was used. The mobile phase was 0.05 M H_2SO_4 in HPLC-grade water, running temperature was 50 , and the flow rate was 0.6 mL min^{-1}. HPLC was used for separation, identifying, and quantifying materials. The gas products were detected using **Gas chromatography (GC)** measurements (Perkin Elmer Clarus 590). Argon (99.999%) was used as the carrier gas. The calibration curve of H_2 representative chromatogram can be found in the Supporting Information, **Figures S9–S10** and **S25**. **Electron paramagnetic resonance (EPR)** spectroscopy was performed at room temperature using a continuous-wave Bruker ELEXSYS E500 CW X-band EPR spectrometer equipped with a standard rectangular high-sensitivity cavity (ER 4119 HS) operating at a microwave frequency of 9.8 GHz. For radical intermediate detection, N-tert-butyl--phenylnitrone (PBN, 50 mM) was employed as the spin trap in dry aprotic DMF. Electrochemical experiments were performed in a divided H-type cell (10 mL) equipped with a standard three-electrode setup, in which the working electrode (bare carbon cloth or UiO-66-CN) and reference electrode (Ag wire quasi-reference) were separated from the Pt foil counter electrode by a Nafion-117 membrane. Freshly prepared electrolyte solutions were thoroughly deaerated and purged with either CO_2 or N_2 prior to each experiment. To probe radical formation, the catholyte was sampled before and after cathodic polarization at -2.24 V vs. SHE. Aliquots of 50 μL were loaded into capillary micropipettes (BRAND GMBH, Germany) and centered within the resonator cavity. Spectra were acquired using a microwave power of 20 mW, a modulation amplitude of 1 G, and a sweep width of 75 G centered at 3518 G. Spectral simulations and hyperfine splitting parameter determinations were performed using the EasySpin software package.

Electrochemical Measurements

All electrochemical measurements were conducted using a 2-compartment H-cell with a three-electrode gas-tight setup in a CO_2-saturated environment, using an Ag quasi reference electrode, and a platinum foil as counter electrode. The 2 cell compartments were separated with an ion-exchange membrane (Nafion 117). Conversion of potential scale to SHE was done using the relation: SHE = -0.662 V vs. $Fc^{+/0}$.[10] Bulk electrolysis measurements were performed at a constant potential, while including an iR-drop compensation extracted using the cell's series resistance. The electrochemical measurements were performed using a BioLogic VSP-300 electrochemical workstation.

Scanning Electrochemical Microscopy (SECM)

Electrode preparation

For the working electrodes preparation, glassy carbon electrode (d= 7mm) was coated with the UiO-66-CN catalyst using a drop-cast method, by depositing 25 L of the UiO-66-CN gel precursor. The electrode was then air-dried for 30 min.

All SECM experiments were performed using a Biologic SECM M470 system using a custom-made four-electrode electrochemical cell, including an Ag wire as a reference electrode, a Pt wire as a counter electrode, and two working electrodes: glassy carbon as a substrate electrode, and an SECM Pt ultramicroelectrode (UME) tip (5 μm radius Pt wire inside 50 μm in radius glass).

Scanning Electrochemical Microscopy (SECM) electrochemical setup.

First, an Electrolyte solution containing 0.1 M $TBAPF_6$ and 1 mM ferrocenemethanol (FcMeOH; 99%) (fast redox agent) in dry DMF was introduced into the SECM cell. A cyclic voltametric (CV) from 0.46 V to 1.06 V (vs. SHE) was performed on the SECM tip at a scan rate of 200 mV/s to confirm the diffusion-limited current and determine the oxidation potential. As shown in **Figure S11**, the limited current was achieved at 0.94 V (vs. SHE) for the oxidation of the Fc complex. Subsequently, a CV from 0.36 V to 1.16 V (vs. SHE) was performed on the glassy-carbon substrate to determine its reduction potential. As shown in **Figure S12**, the limited current was achieved at 0.31 V (vs. SHE) for the reduction of the Fc complex. Next, to determine the tip-to-substrate distance, an approach curve experiment toward the glassy-carbon substrate was performed by applying a constant potential of 0.94 V on the tip and a constant potential on the substrate of 0.31 V (both vs. SHE). The SECM tip was moving towards the substrate electrode surface at a rate of 1 μm/sec. The recorded data was fitted to the theoretical model (SECM conductor) using MIRA simulation software to accurately determine the tip-to-substrate distance (**Figure S13**). Thereafter, the tip was retracted upward, and the bare glassy-carbon substrate was coated with a 9.5 μm thick UiO-66-CN layer (as measured using SEM-FIB, see **Figure S14**). To ensure high collection efficiency and allow detection of the electrochemically formed $CO_2^{-•}$, the tip was then placed within the MOF layer by approaching it to a tip-to-substrate distance of 5 μm, meaning that the tip was approximately 4.5 μm inside the MOF layer.

ATR-IRRAS (Attenuated total reflectance-Infrared reflection absorption spectroscopy in Otto configuration)

Electrode preparation

The glassy carbon electrode (d= 6mm) was prepared using the drop-cast method by depositing 10 μL of the UiO-66-CN. The electrode was then dried for 10-20min.

ATR measurements were recorded using the Thermo Nicolet iS50 instrument equipped with liquid nitrogen cooled MCT-A detector using ZnSe window (4000 - 650 cm^{-1}). A custom-made spectro-electrochemical cell supplied by the Beijing Scistar technology (Co. Ltd.) was mounded on PIKE instrument VeeMAXIII ATR optical accessory. A circular ZnSe disc window was used for all the ATR measurements. All the IR data were recorded and processed using the software OMNIC SPECTRA, supplied by Thermo Fischer Scientific.

Experimental details of ATR-IRRAS (Attenuated total reflectance-Infrared reflection absorption spectroscopy in Otto configuration)

The electrochemical cell consisted of a 3-electrode set-up, where the working electrode sat tightly above the ZnSe window, with the help of an O-ring. The reference and counter electrodes were placed nearby but were not exposed to the incident IR beam. The UiO-66-CN catalyst coated on glassy carbon electrodes (5μL) of a few microns thickness were used as the working electrodes, Ag wire electrode was used as the reference electrode, and a Pt foil was employed as the counter electrode for all measurements. A thin solution layer was present between the catalyst coated GC electrode and the ZnSe window. The thin layer solution was exposed to the incident IR light. The working electrode surface and the thin layer solution between the IR window and working electrode were studied by the ATR-IRRAS measurement. Analyte or electrolyte solution could be continuously flowed within the cell by solution inlet and outlet using a pump connected to a stock reservoir. For all the ATR-IRRAS measurements, 0.1 M $TBAPF_6$ in dry DMF was first purged with Ar for 40 minutes and flowed through the cell with a flow rate of 15 ml/min and then background was collected before starting the experiments analysis.

Supporting Information

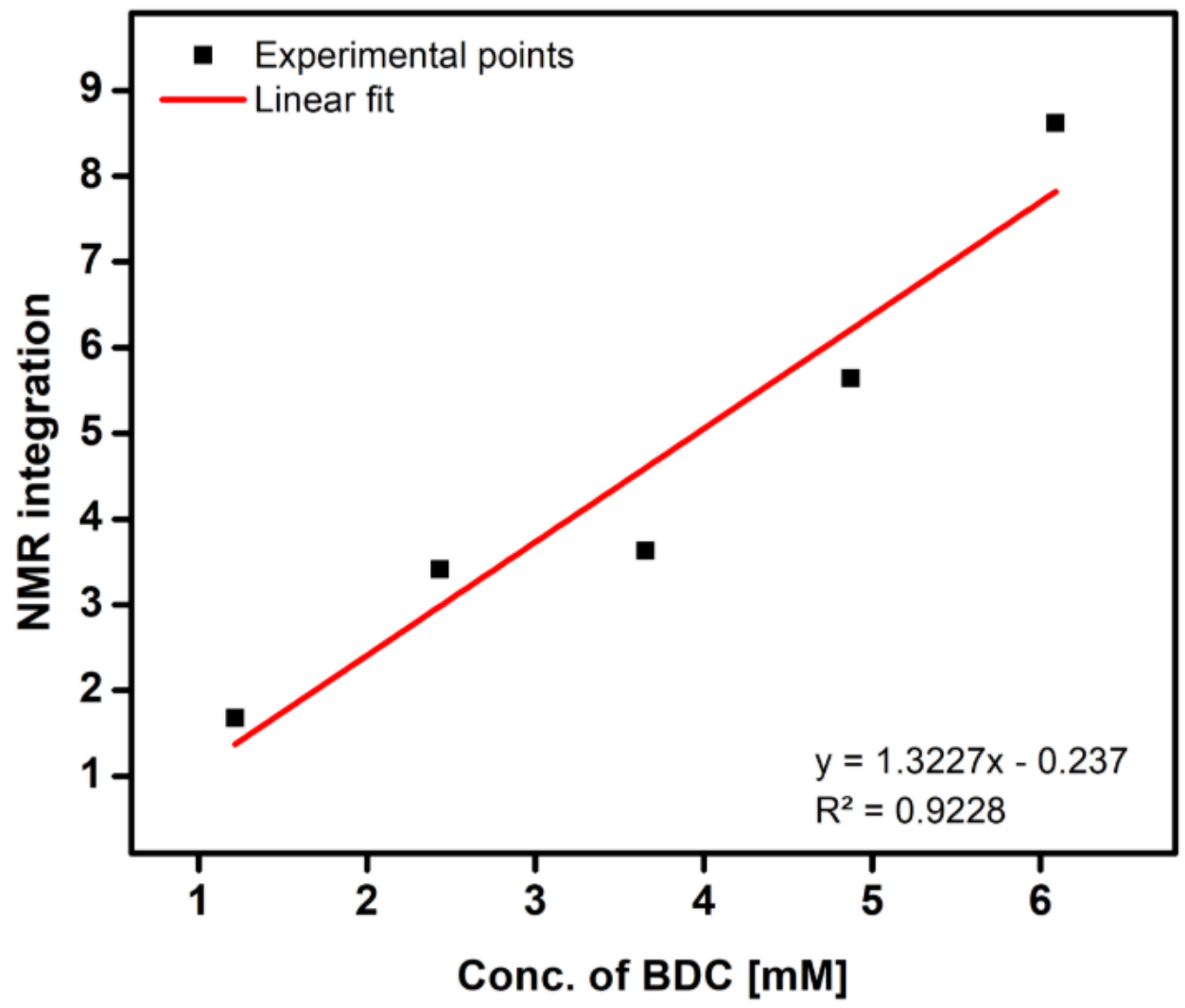

Figure S1. ^{1}H NMR calibration plot to determine the amount of **benzene-1,4-dicarboxylic acid** (BDC) in UiO-66 and UiO-66- CN gel.

Table S1. ICP-OES of UiO-66 and UiO-66-CN.

Sample	m [mg]	gr/L	V [L]	mol of Zr	mol of Zr in 1mg	mol of Zr_6	mol of BDC	BDC/Zr_6	4-CB/ Zr_6
UiO-66-CN	2.4	0.005256	0.0098	4.84 ∙ 10^{-6}	2.02 ∙ 10^{-6}	3.36 ∙ 10^{-7}	1.81 ∙ 10^{-6}	5.39	2.65
UiO-66	2.1	0.005174	0.0098	4.76 ∙ 10^{-6}	2.27 ∙ 10^{-6}	3.78 ∙ 10^{-7}	1.54 ∙ 10^{-6}	4.07	-

Quantifying the density of missing linker defect in UiO-66 and loading of 4-cyanobenzoic acid (4-CB) groups in UiO-66-CN with the help of ICP-OES and ^{1}H-NMR.

ICP-OES analysis: a known amount of UiO-66 gel was digested in 6 mL (V_d) of concentrated HNO_3 at 120 ℃ for 12 hours. 0.7 mL (V_i) of the acid mixture was diluted to 9.8 mL (V_f) by adding water and analyzed by ICP-OES.

From the experimentally obtained concentration of Zr ions in the 9.8 mL solution, the concentration of Zr_6 nodes was calculated.

The density of Zr in UiO-66 was analyzed from ICP to be = 5.174 mg/L.

2.1 mg of UiO-66 contains = $ICP_{density}$ ∙ V_f ∙ $\frac{V_d}{V_i}$ = 0.4346 mg of Zr.

Thus, 2.1 mg of UiO-66 contains 4.76×10^{-6} moles of Zr.

Thus, 1 mg of UiO-66 contains 2.27×10^{-6} moles of Zr.

Thus, 1 mg of UiO-66 contains 3.78×10^{-7} moles of Zr_6.

Similarly, for UiO-66-CN, the density of Zr in UiO-66-CN was analyzed from ICP to be = 5.256 mg/L.

Thus, 2.4 mg of UiO-66 contains 0.4415 mg of Zr.

Thus, 2.4 mg of UiO-66-CN contains 4.84×10^{-6} mole of Zr.

Thus, 1 mg of UiO-66-CN contains 2.02×10^{-6} mole of Zr.

Thus, 1 mg of UiO-66-CN contains 3.36×10^{-7} mole of Zr_6.

Now, the amount of BDC in 1 mg UiO-66 = 1.54×10^{-6} mole.

Considering the results obtained from ICP-OES analysis for UiO-66 gel, BDC/Zr6 = 4.07.

Thus, the number of BDC per node = 8.15 ≈ 8 for UiO-66 gel.

So, number of defect sites per node = (12 – 8) = 4 for UiO-66 gel.

Amount of BDC in 1 mg UiO-66-CN = 1.81×10^{-6} mole.

Considering the results obtained from ICP-OES analysis for UiO-66-CN, BDC/Zr6 = 5.39.

Thus, the number of BDC per node should be 10.8 for UiO-66-CN gel.

The excess BDC of (10.8 – 8.15) = 2.65 ≈ 3 per Zr_6-oxo node of UiO-66-CN is a result of the hydrolyzed 4-CB groups in addition to the already present BDC groups. The –CN group cannot withstand the high basic environment of 2 M NaOH, which was used to digest the MOF into an aqueous solution. The –CN groups get hydrolyzed to –COOH groups under the condition of digestive NMR sample preparation.

Thus, the loading level of 4-CB was ≈ 3 per Zr_6-oxo node.

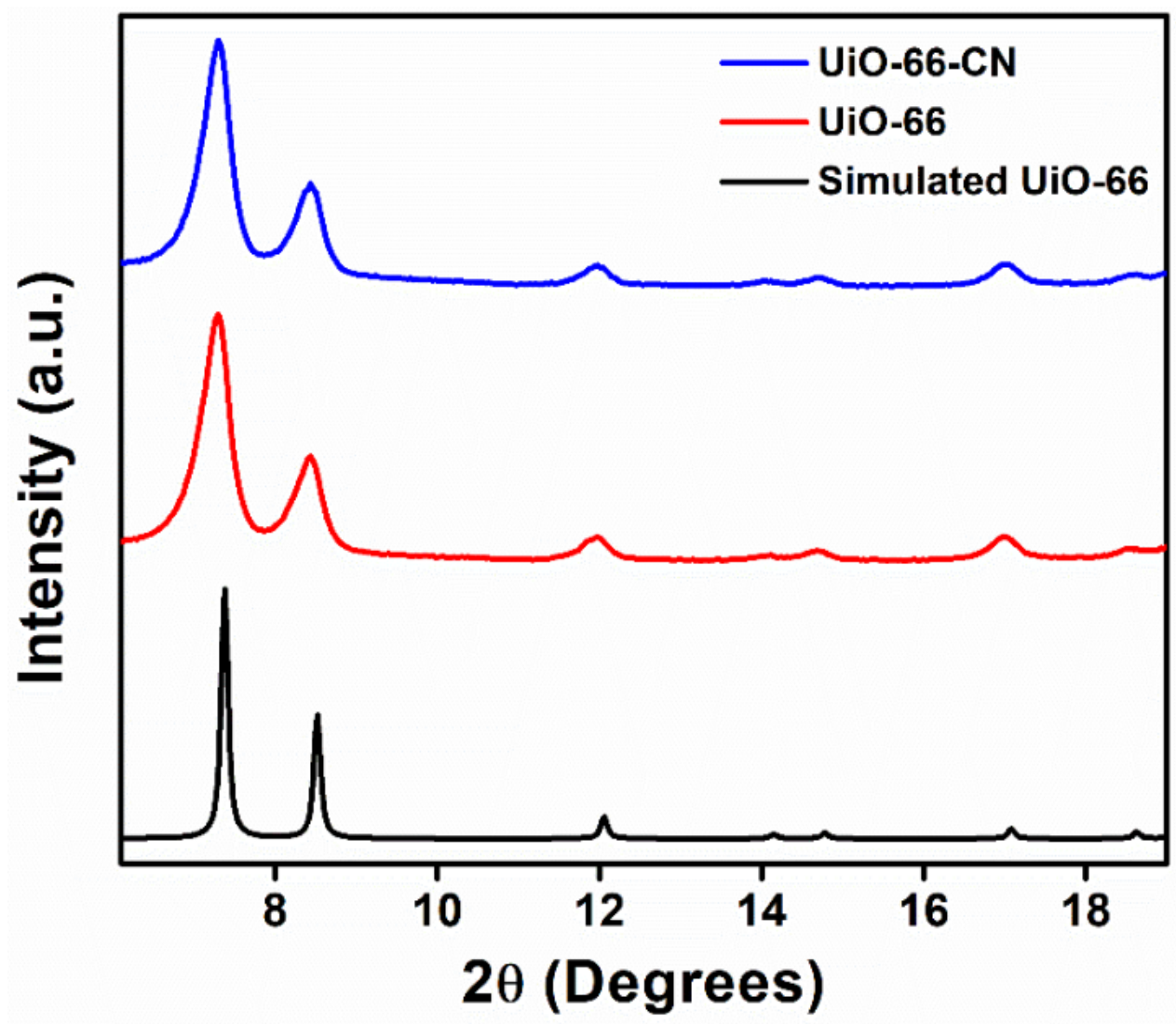

Figure S2. XRD measurement of UiO-66 powder (red), UiO-66-CN powder (blue) and simulated UiO-66 (black).

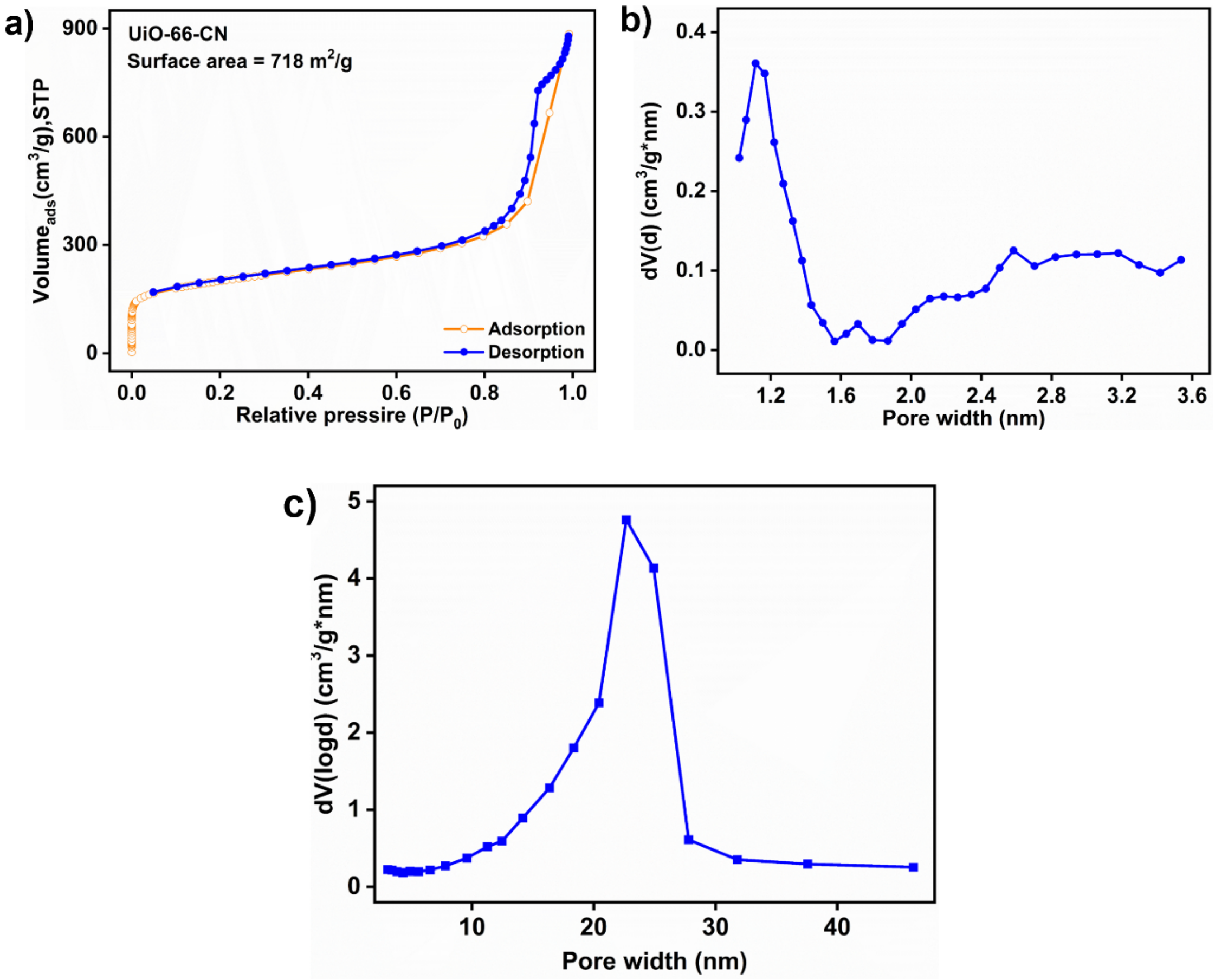

Figure S3. a) N_2 iso physisorption isotherms of UiO-66-CN and the surface areas of the MOF, calculated from BET. b) The micropore size distribution for the UiO-66-CN. c) The mesopore size distribution for the UiO-66-CN.

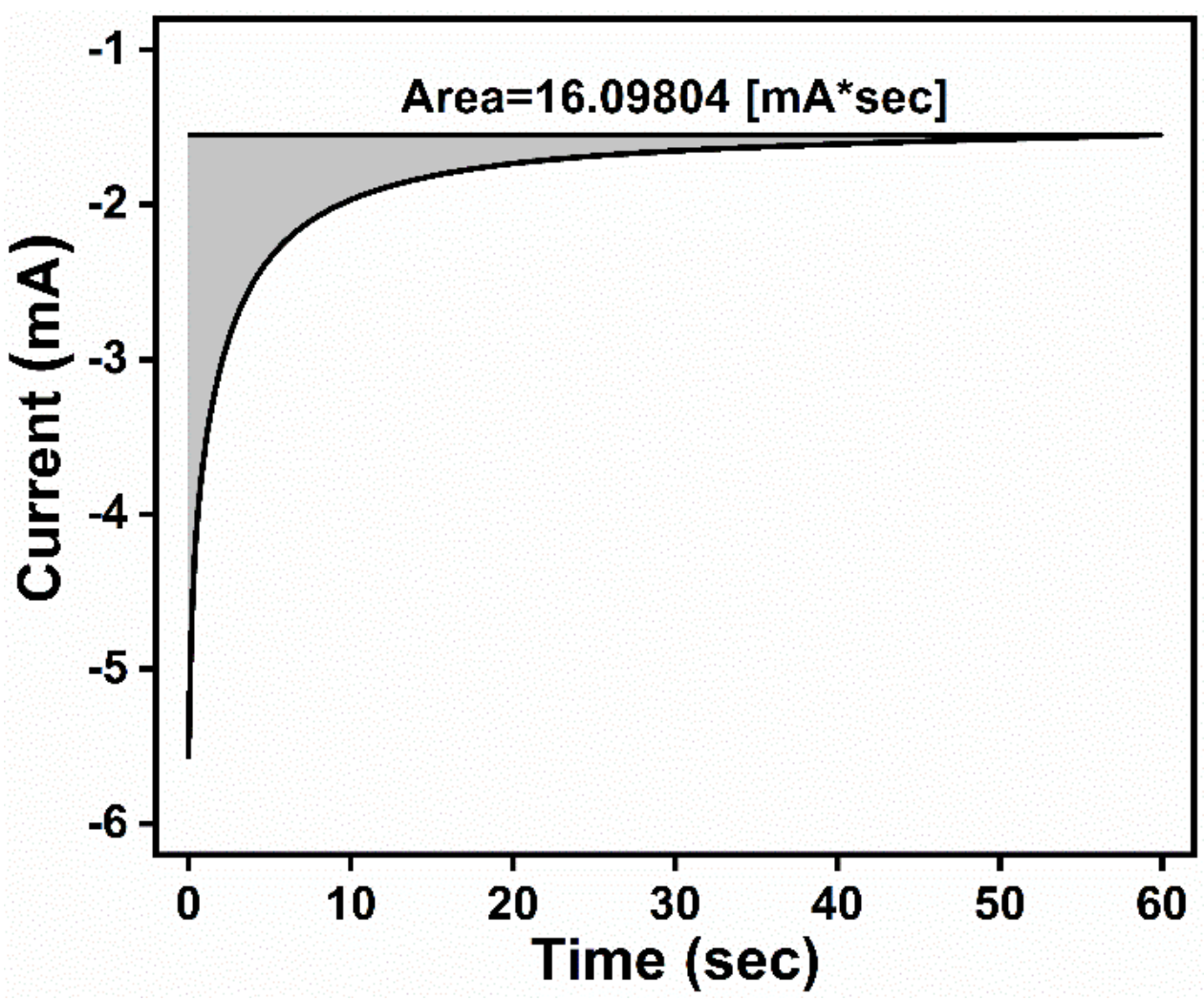

Figure S4. Electroactive moles of 4-CB in UiO-66-CN using potential-steps chronoamperometry (-2.44V vs. SHE).

Quantifying the number of active sites in UiO-66-CN

$$\text{Total loaded active site} = n_{total} = \frac{m(\text{dry UiO - 66 - CN})}{MW(\text{UiO - 66 - CN})}$$

$$\text{Molar mass (UiO - 66 - CN)}\left[\frac{gr}{mol}\right]$$

== Molar mass (UiO - 66) - Molar mass (BDC) * number of defects per node + Molar mass (4 - CB) * CN /node molar ratio

$$\text{Molar mass (UiO - 66 - CN)}\left[\frac{gr}{mol}\right] = 1664.06\left[\frac{gr}{mol}\right] - 166.13\left[\frac{gr}{mol}\right] * 2 + 147.13\left[\frac{gr}{mol}\right] * 2.65 = 1721.64\left[\frac{gr}{mol}\right]$$

Mass of UiO - 66 - CN after drying [gr] = 0.0004 [gr] in 100 [μL]

$$\text{Total loaded active site} = n_{total} = \frac{m(\text{dry UiO - 66 - CN})}{MW(\text{UiO - 66 - CN})} = \frac{0.0004[gr]}{1721.64[\frac{gr}{mol}]} = 2.323 * 10^{-7}\ [mol]$$

$$\text{Electroactive moles} = n_{electroactive} = \frac{\text{Area } [A * sec]}{F\ [\frac{A * sec}{mol}]}$$

Quantifying the electroactive moles in UiO-66-CN using potential-steps chronoamperometry analysis (Figure S4).

Area from the potential steps chronoamperometry analysis = 0.0161 [A * sec]

$$\text{Electroactive moles} = n_{electroactive} = \frac{0.0161\ [A * sec]}{96485.3383\ [\frac{A * sec}{mol}]} = 1.668 * 10^{-7}\ [mol]$$

The electroactive to total active site fraction (%)

$$\frac{n_{electroactive}}{n_{total}} = \frac{1.668 * 10^{-7}\ [mol]}{2.323 * 10^{-7}\ [mol]} * 100\% = 71.8\ \%$$

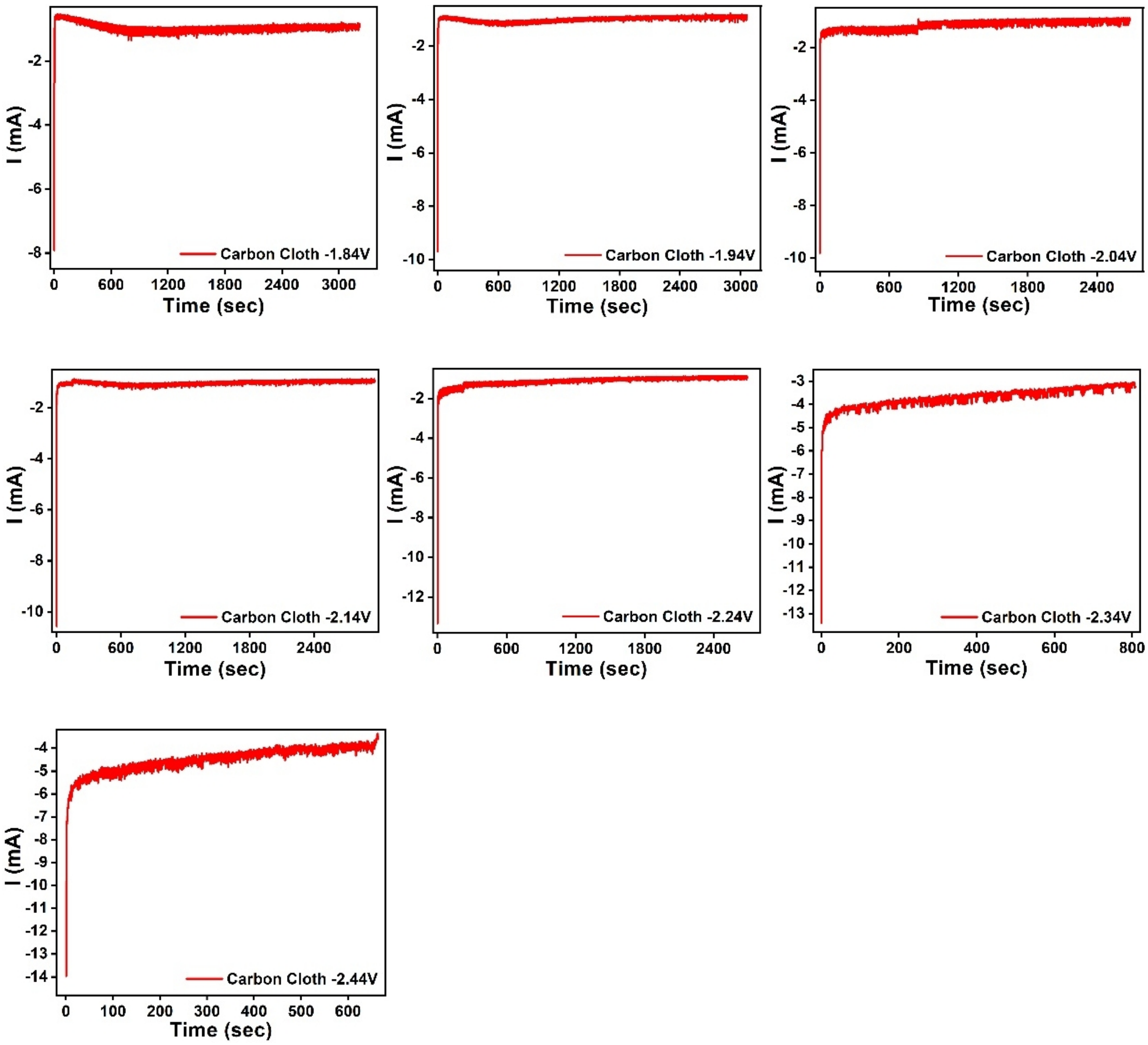

Figure S5. chronoamperometry measurements at various potentials (vs. SHE) for oxalate formation on carbon cloth (CC).

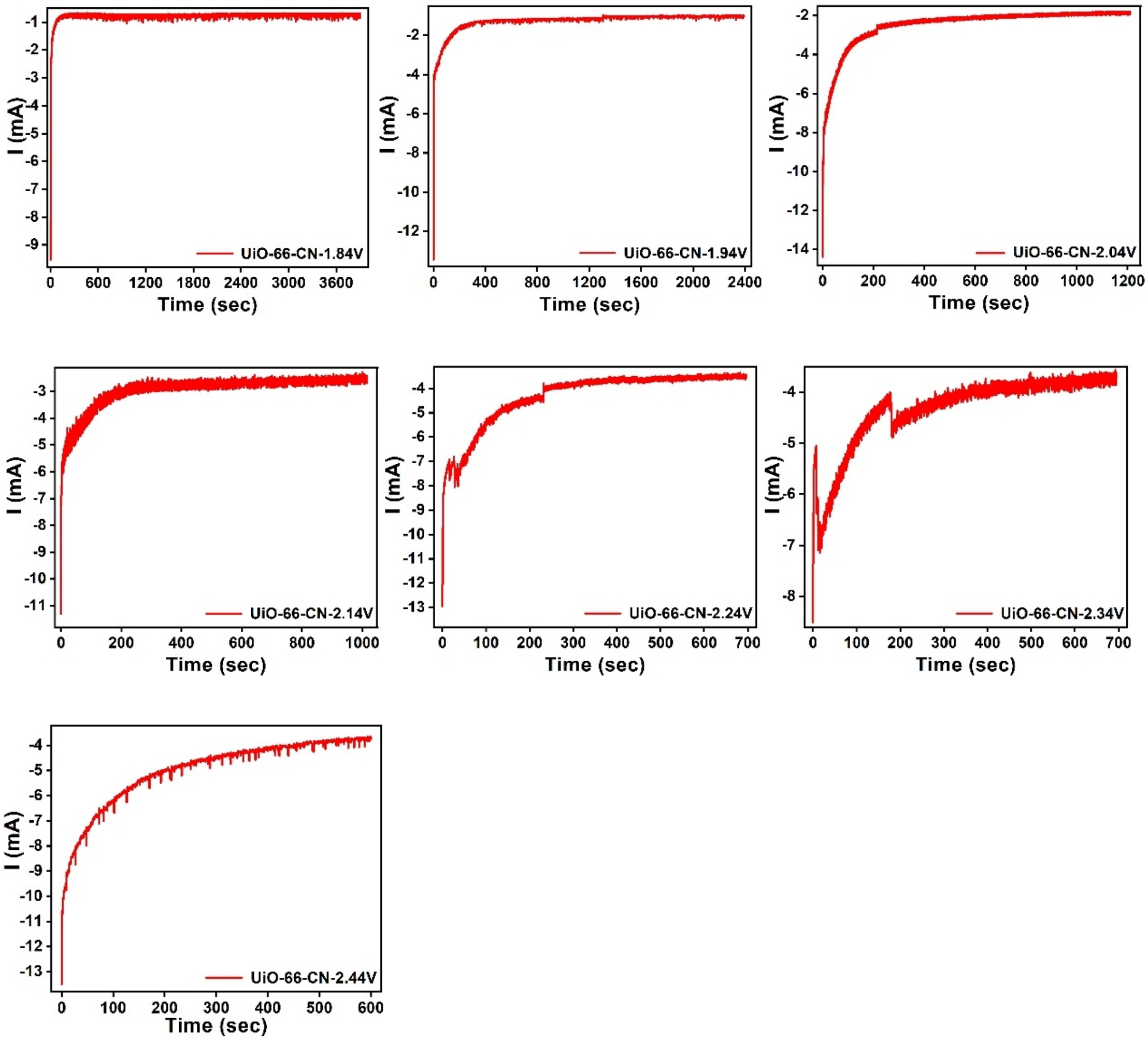

Figure S6. chronoamperometry measurements at various potentials (vs. SHE) for oxalate formation on CC-UiO-66-CN.

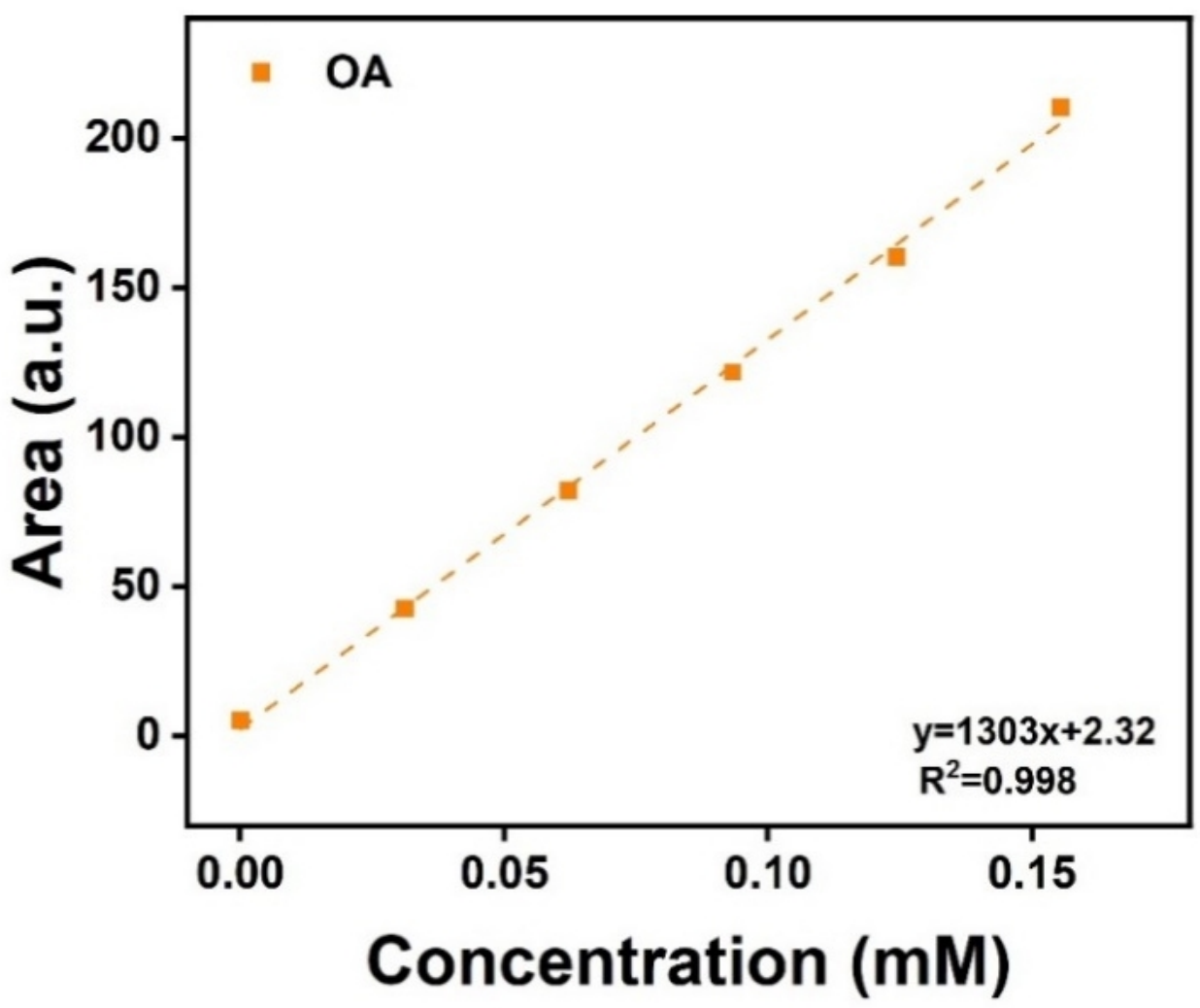

Figure S7. HPLC calibration plot for oxalic acid.

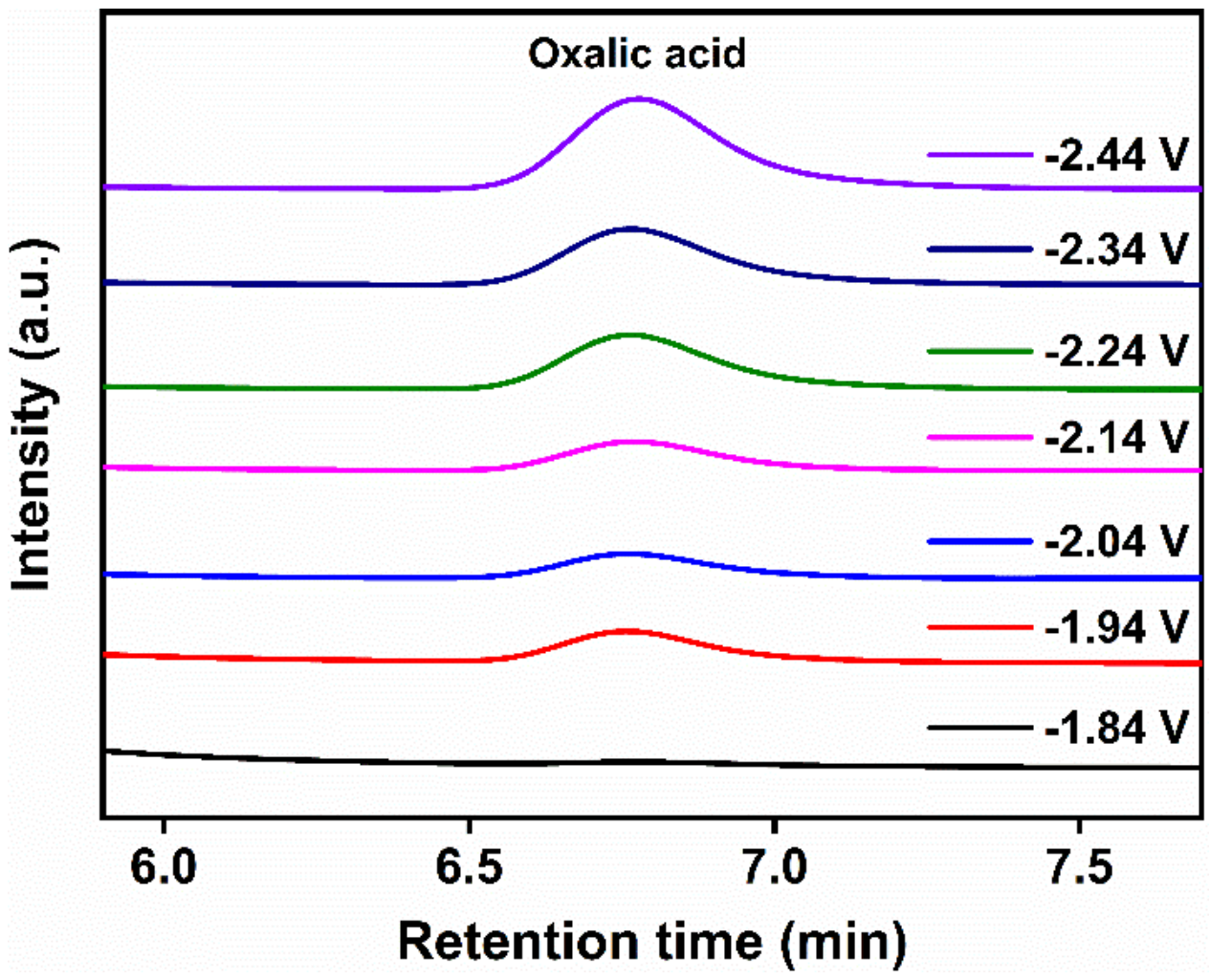

Figure S8. HPLC of UiO-66-CN electrode with typical retention time for Oxalic acid detaction at different potential ..

Calculation of oxalic acid from HPLC:

For example, the area under the graph of oxalic acid at -2.44 V vs. SHE is: **176**

Using the calibration curve equation and the area that was obtained from the HPLC we get the concentration of oxalic acid in the solution.

((176-2.32)/1303)*10 = **1.33mM**

(10 indicate dilution retio before injection).

Next, we divide the concentration by 1000 to convert it to M, and then divide by the total volume of the cell (10 mL).

(1.33mM/1000)*0.01L = **1.33E-5 mol**

The total electron of the reaction (2 e^- reaction, 3C charge)resulting **1.55E-5 mol**

%FE= (1.33E-5 mol/1.55E-5 mol)*100 ≈ **85%**

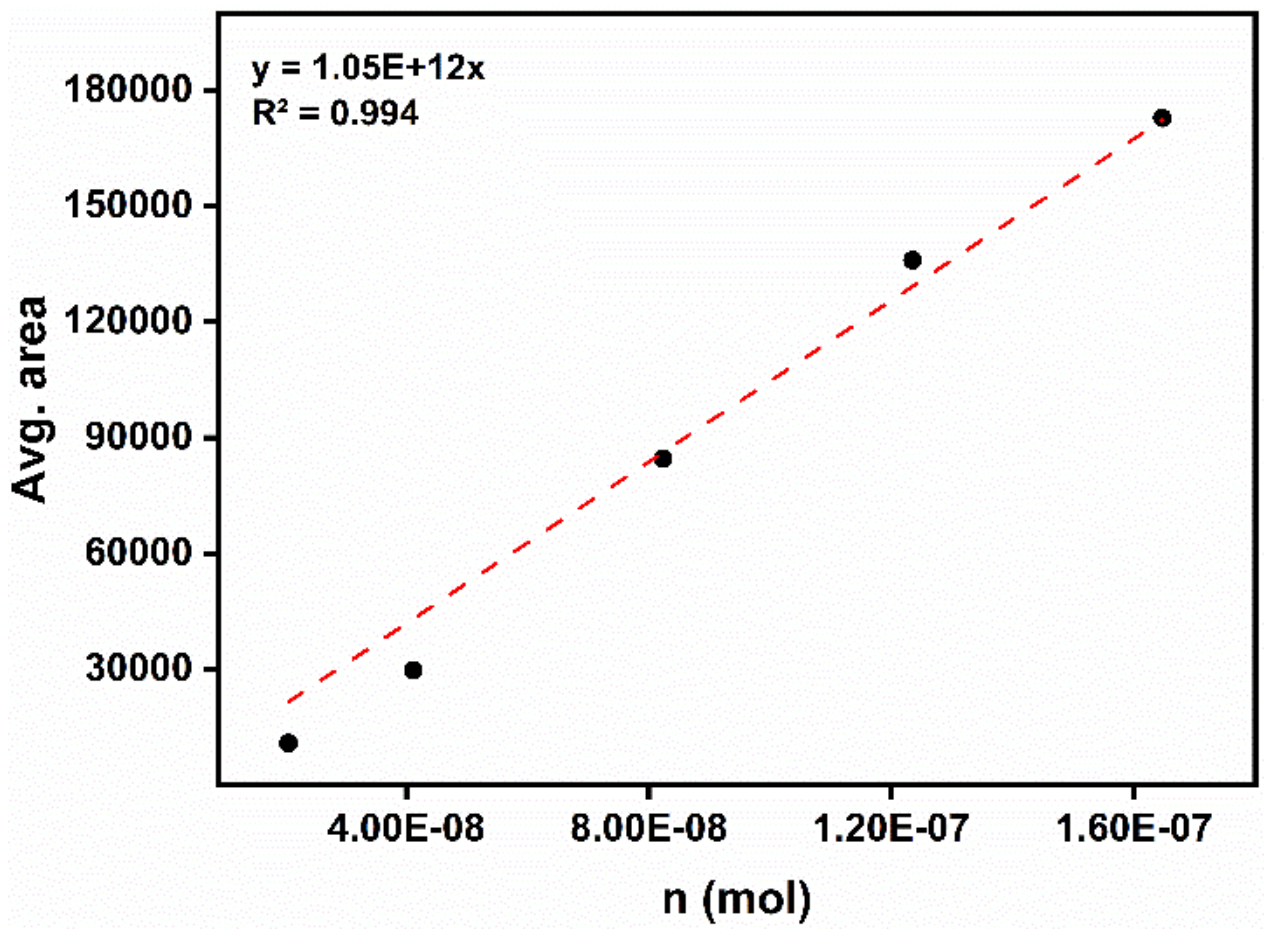

Figure S9.gas chromatograpy Calibration curve for H_2 .

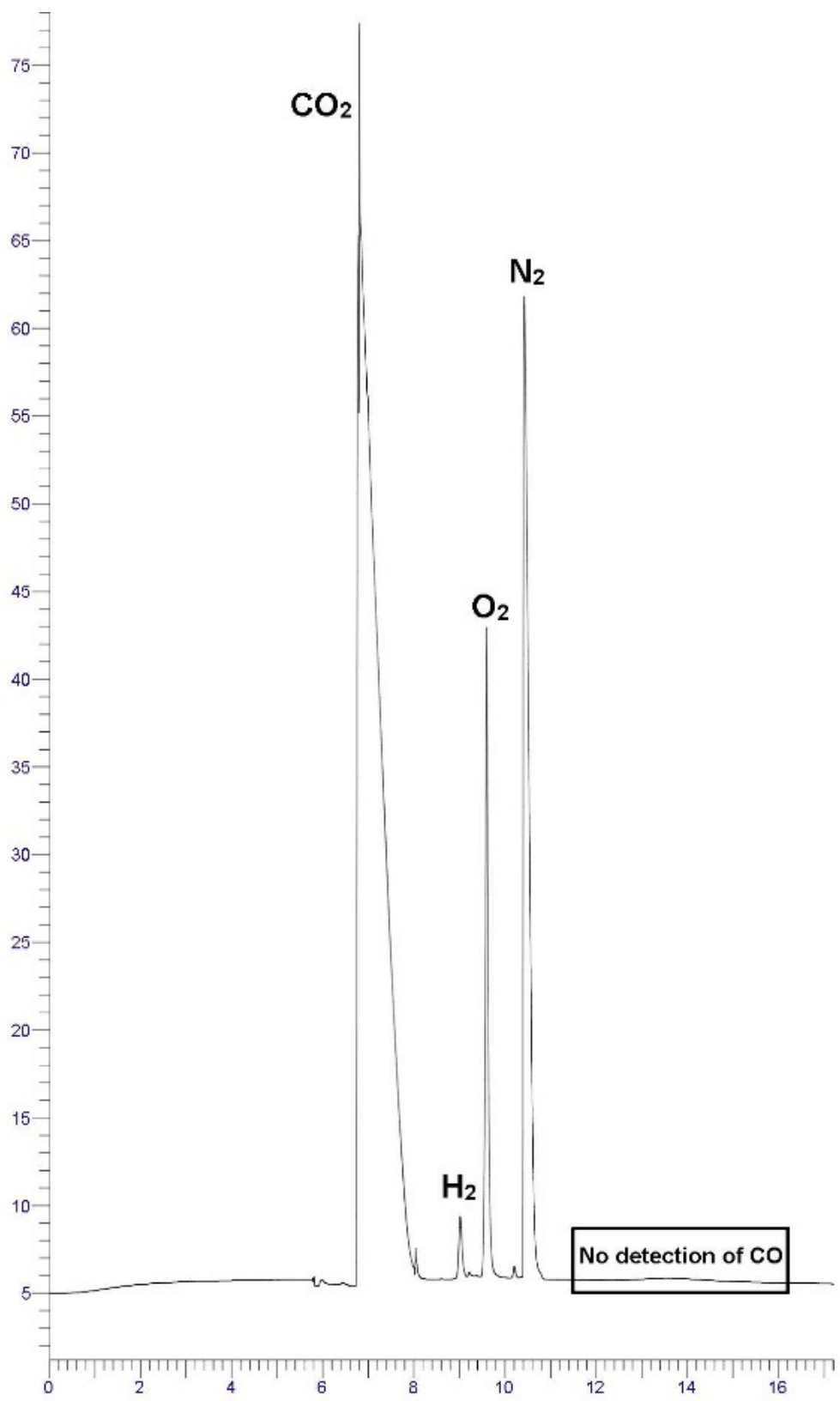

Figure S10. GC chromatogram of H_2 for UiO-66-CN in CO_2 experiment at -2.44 V vs. SHE

Calculation of H_2 from GC:

For example, the area under the graph of H_2 at -2.44 V vs. SHE is: **17994**

Using the calibration curve equation and the area that was obtained from the GC we get the mol number of H_2 in the 0.5 mL syringe.

(17994/1.05E+12) = **1.714E-8 mol**

The total head space is 58 mL so we multiple by 116 to get the total mol number of H_2 in the cell.

1.714E-8 mol * (58 mL/0.5mL) = **1.988E-6 mol**

The total electron of the reaction (2 e^- reaction, 3C charge) is **1.55E-5 mol**

%FE= (1.988E-6 mol/1.55E-5 mol)*100 ≈ **13%**

Thus, the percentages of FE towed H_2 is 13%

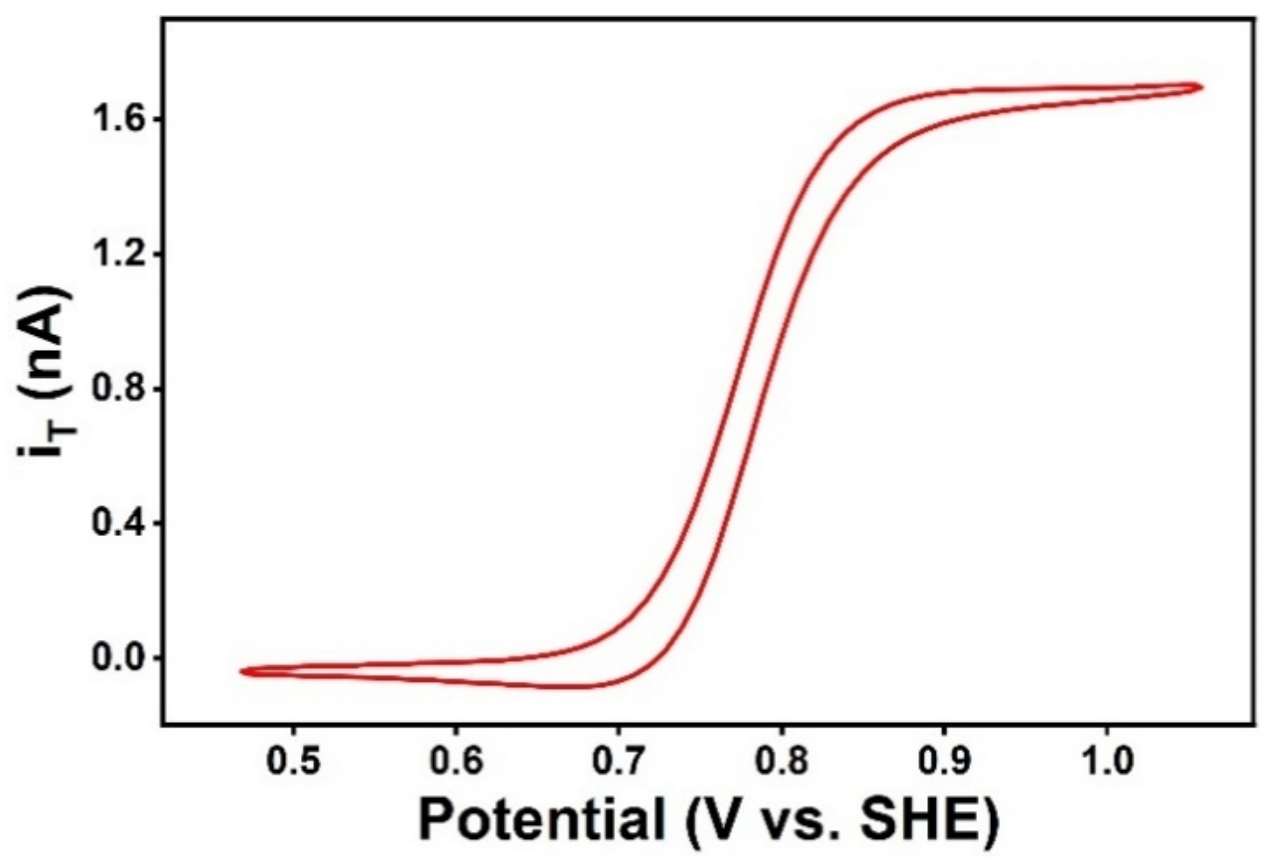

Figure S11. CV of 1 mM ferrocenemethanol (FcMeOH) in dry DMF containing 0.1M $TBAPF_6$ at a a = 5 μm Pt UME tip. The scan rate was 200 mV·s−1.

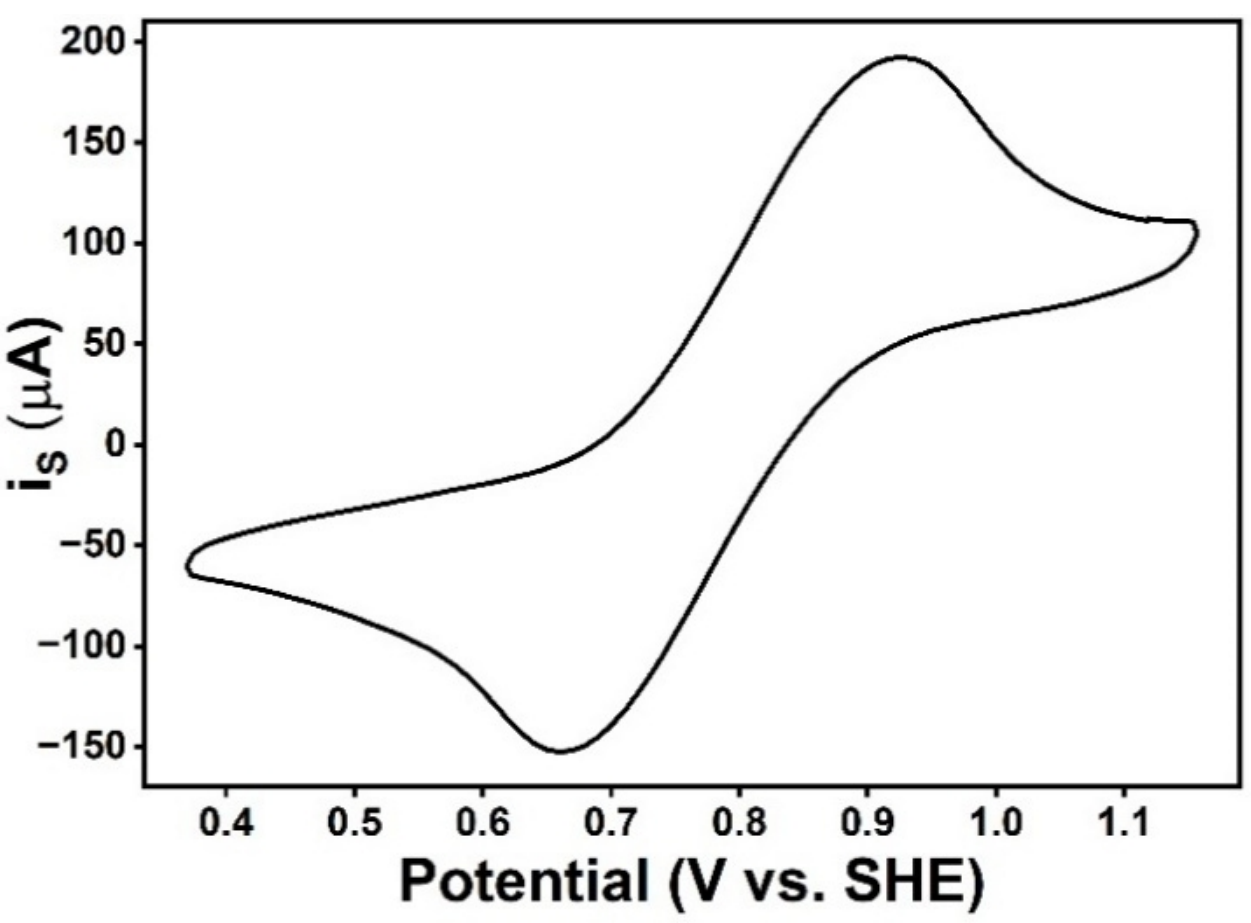

Figure S12. CV of 1 mM ferrocenemethanol (FcMeOH) in dry DMF containing 0.1M $TBAPF_6$ at a glassy carbon substrate. The scan rate was 200 $mV·s^{-1}$.

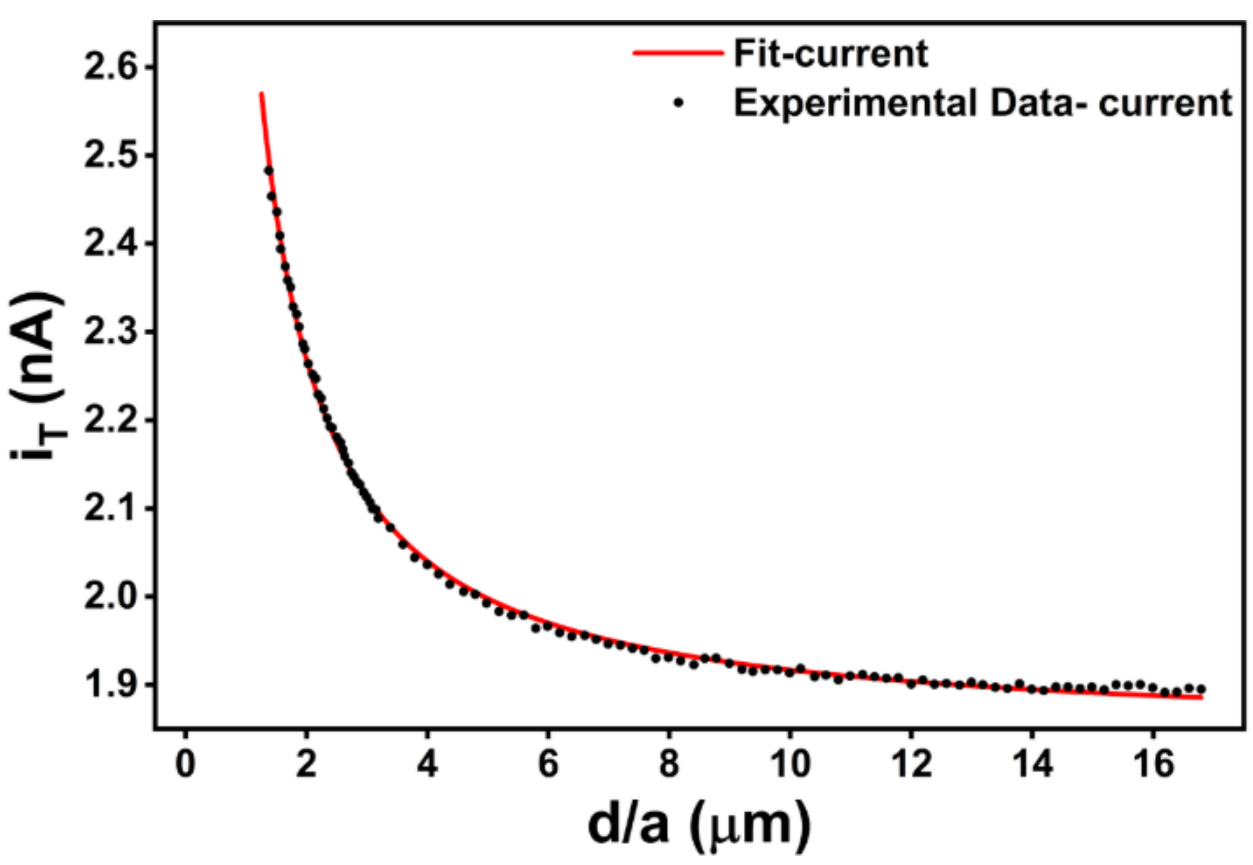

Figure S13. Positive feedback approach curve (Black dotted curve) for 1 mM FcMeOH in dry DMF containing 0.1 M $TBAPF_6$ obtained with a a = 5 μm Pt UME tip (SECM tip, RG = 10) and a glassy carbon surface (SECM substrate). The tip and substrate potentials were held at 0.94 V and 0.31 V, respectively (vs. SHE). A theoretical approach curve (Red curve) was obtained from MIRA simulation.

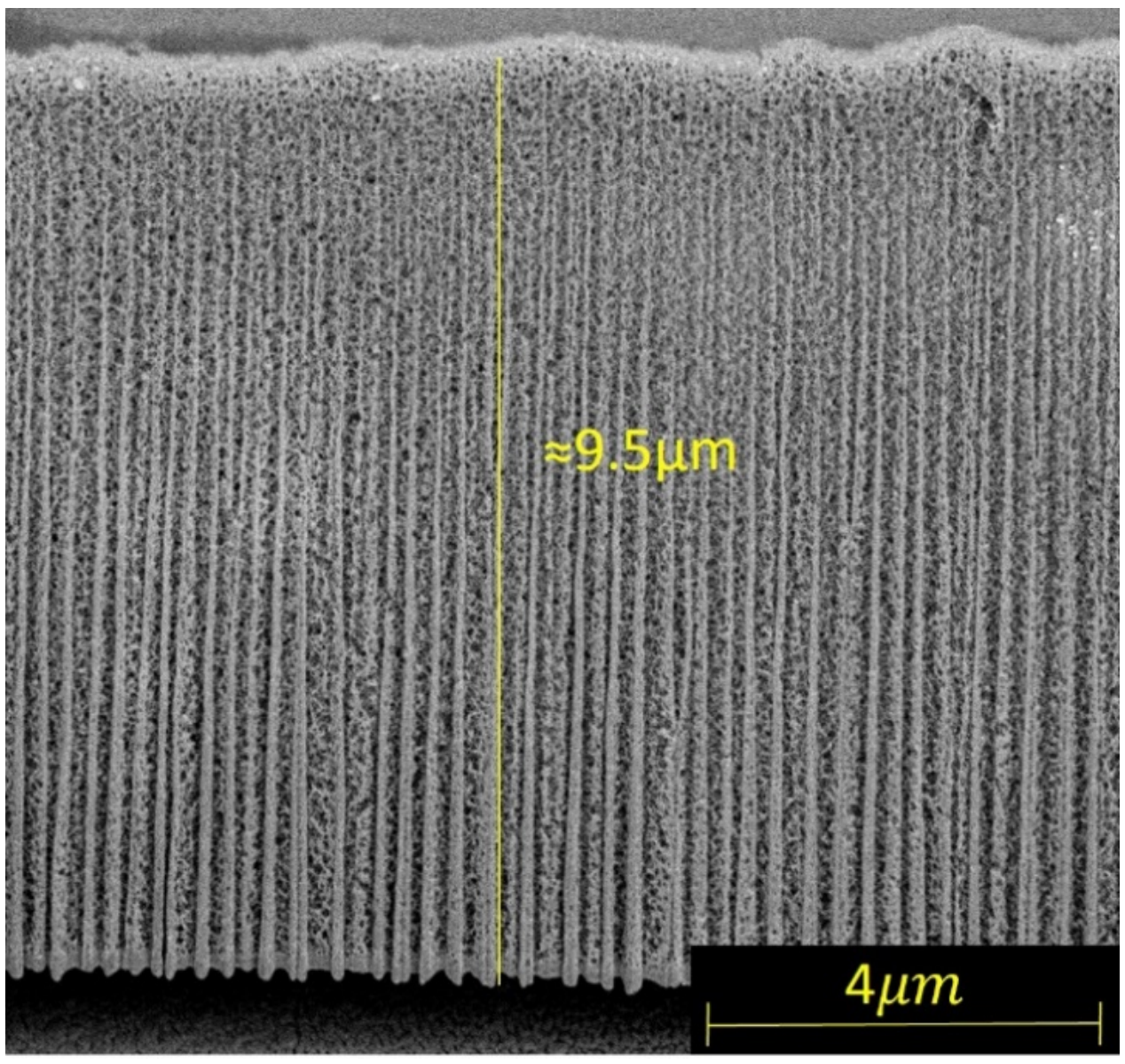

Figure S14. SEM-FIB cross-section images of UiO-66-CN on glassy carbon electrode as used in SECM experiments.

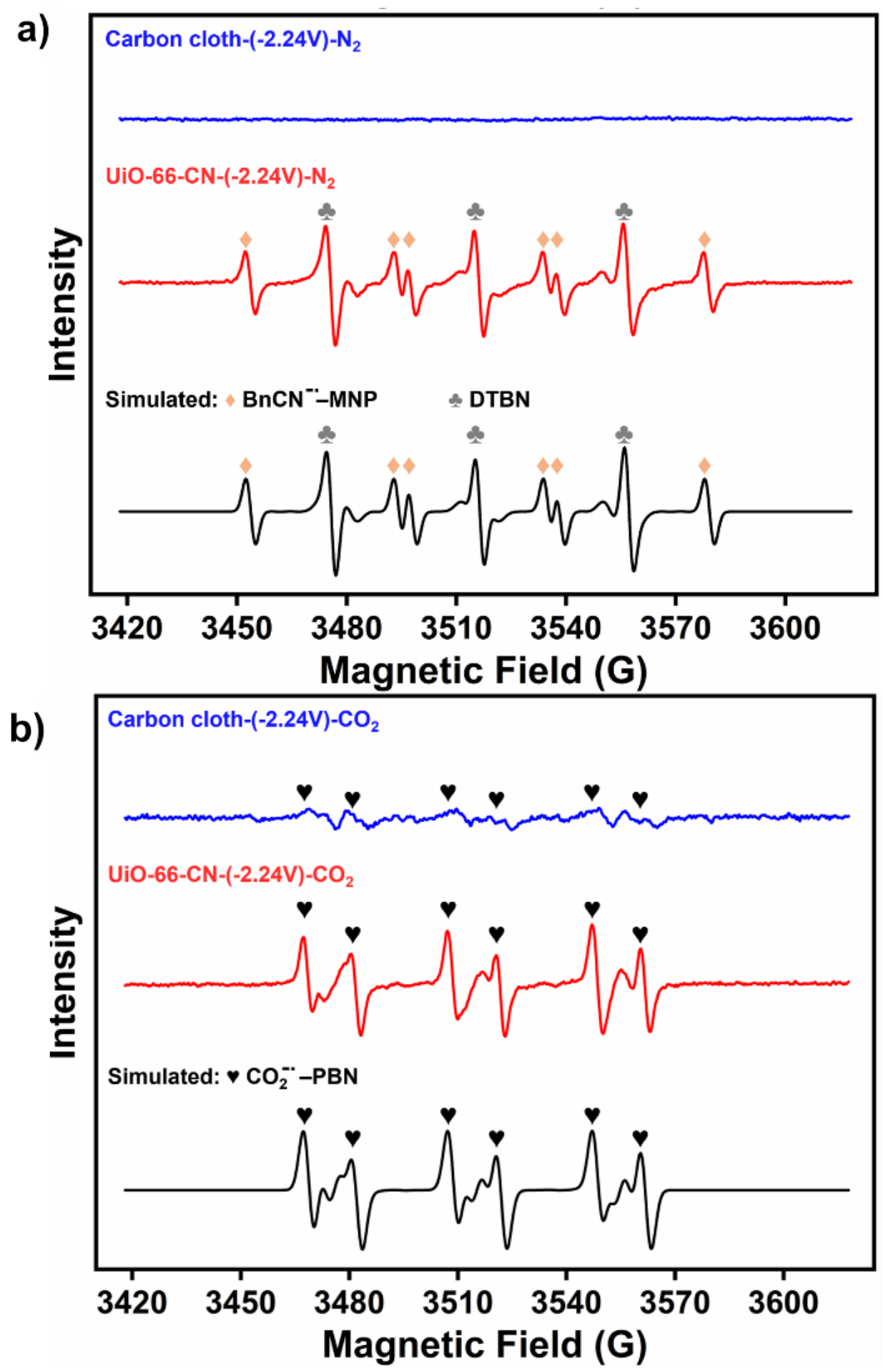

Figure S15. EPR spectra of the catholyte solution following electrolysis in an H-cell at -2.24 V vs. SHE under (a) N_2 and (b) CO_2 atmospheres, comparing bare carbon cloth and UiO-66-CN alongside spectral simulations. Characteristic signals for the trapped radical species ($CO_2^{-\bullet}$ and $BnCN^{-\bullet}$) and spin-trap derivatives are indicated by symbols.

Ex situ EPR spin-trapping: measurements were conducted on the catholyte solution following electrolysis at -2.24 V vs. SHE to directly probe transient radical intermediates via a N-tert-Butyl-α-phenylnitrone (PBN) spin trap (**Figure S15**). To facilitate the detection of framework-tethered species, vigorous gas purging was directed adjacent to the electrode surface to deliberately promote the detachment of a minor quantity of UiO-66-CN into the bulk catholyte suspension. Under an inert N_2 atmosphere, the UiO-66-CN electrode displayed a distinctive multi-line pattern arising from the superposition of a triplet and a split quartet, while bare carbon cloth showed a silent spectrum (**Figure S15a**). Under these applied cathodic potential, partial transformation of PBN yields secondary trapping species such as 2-methyl-2-nitrosopropane (MNP)[7, 8] and di-*tert*-butyl nitroxide (DTBN)[9]. The existence of a triplet (aN = 15.3 G) in the UiO-66-CN spectrum is a characteristic of a such DTBN radical (or related symmetric nitroxide), while the split quartet (aN = 15.3 G, aN = 8.3 G) fits an $BnCN^{-\bullet}$-MNP adduct formed via reaction with the electrogenerated benzonitrile radical species. Under a CO_2 atmosphere, the bare carbon cloth electrode produced only a negligible background response, whereas the UiO-66-CN electrode generated a prominent, well-resolved six-line spectrum (**Figure S15b**).[5, 6] Spectral simulation yielded hyperfine splitting constants of aN = 15.0 G and aH = 5.0 G, matching the established signature of the $CO_2^{-\bullet}$-PBN spin adduct and confirming framework-promoted single-electron activation of CO_2 (**Figure S15b**). Notably, signals related to secondary trap-derived species (such as DTBN or MNP adducts) were not observed in the presence of CO_2. This is likely due to favorable reaction kinetics under CO_2-saturated conditions, where rapid radical capture preferentially yields the persistent $CO_2^{-\bullet}$-PBN adduct and suppresses the accumulation of other detectable radical species. Hence, the spectral simulations in Figure S15 show excellent agreement with the experimental data, providing definitive spectroscopic verification for the electrogeneration of both the framework-tethered $BnCN^{-\bullet}$and the resulting $CO_2^{-\bullet}$ radical intermediates.

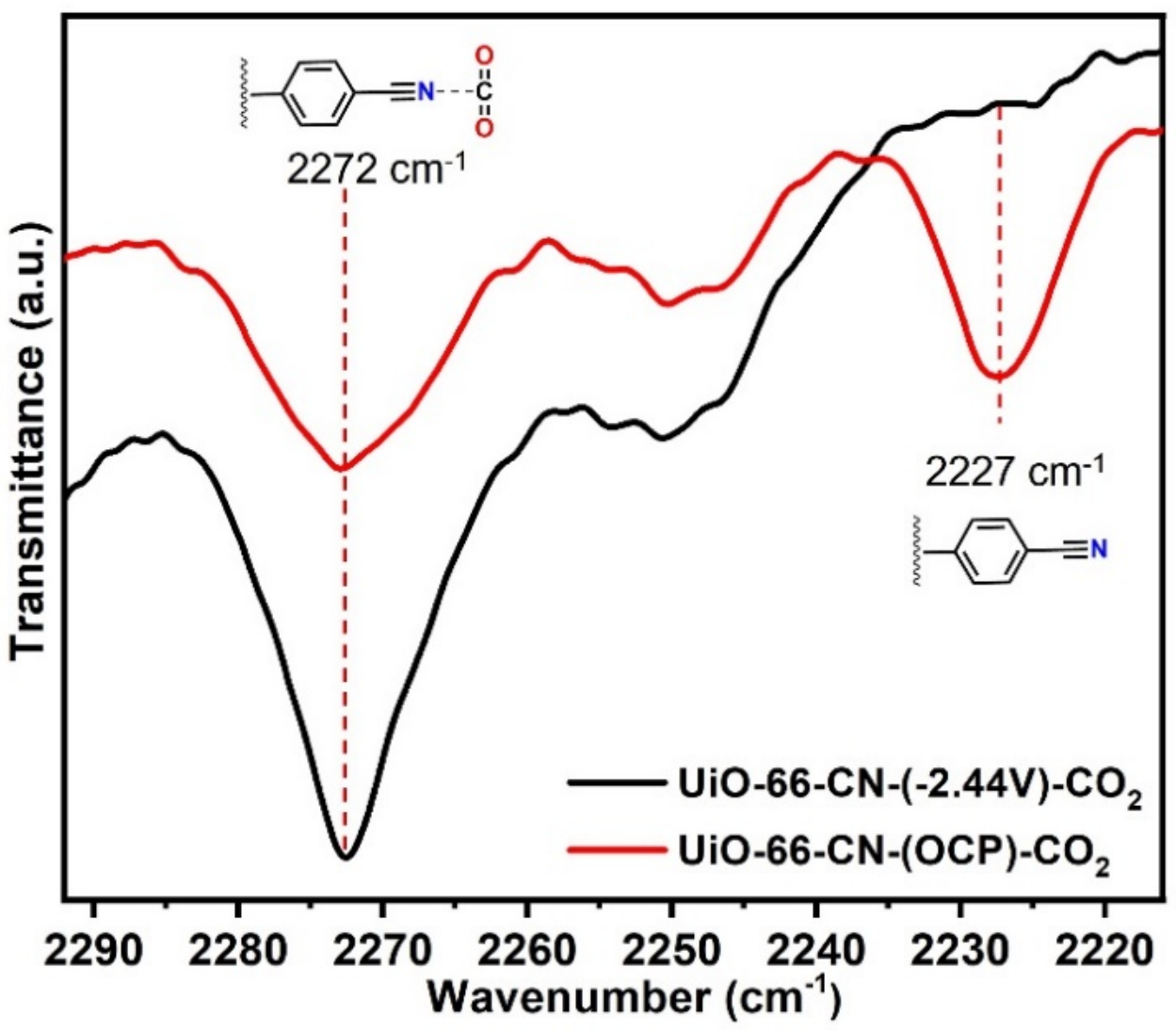

Figure S16. Operando FTIR measurement of UiO-66-CN under OCP (red) and under -2.44V vs. SHE (black) with CO_2.

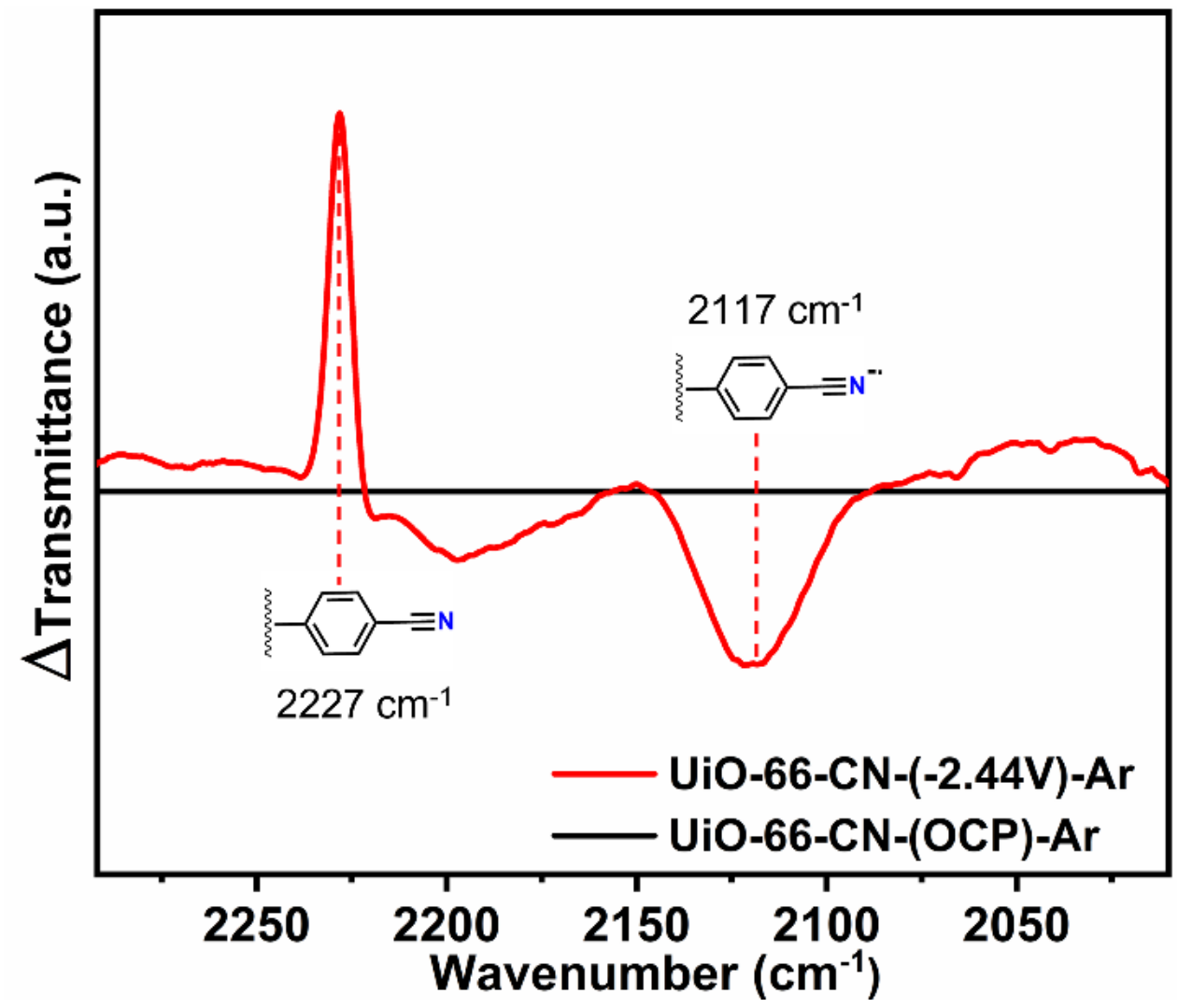

Figure S17. Operando FTIR measurement of UiO-66-CN under OCP (red) and under -2.44V vs. SHE (black) with Ar after OCP subtraction.

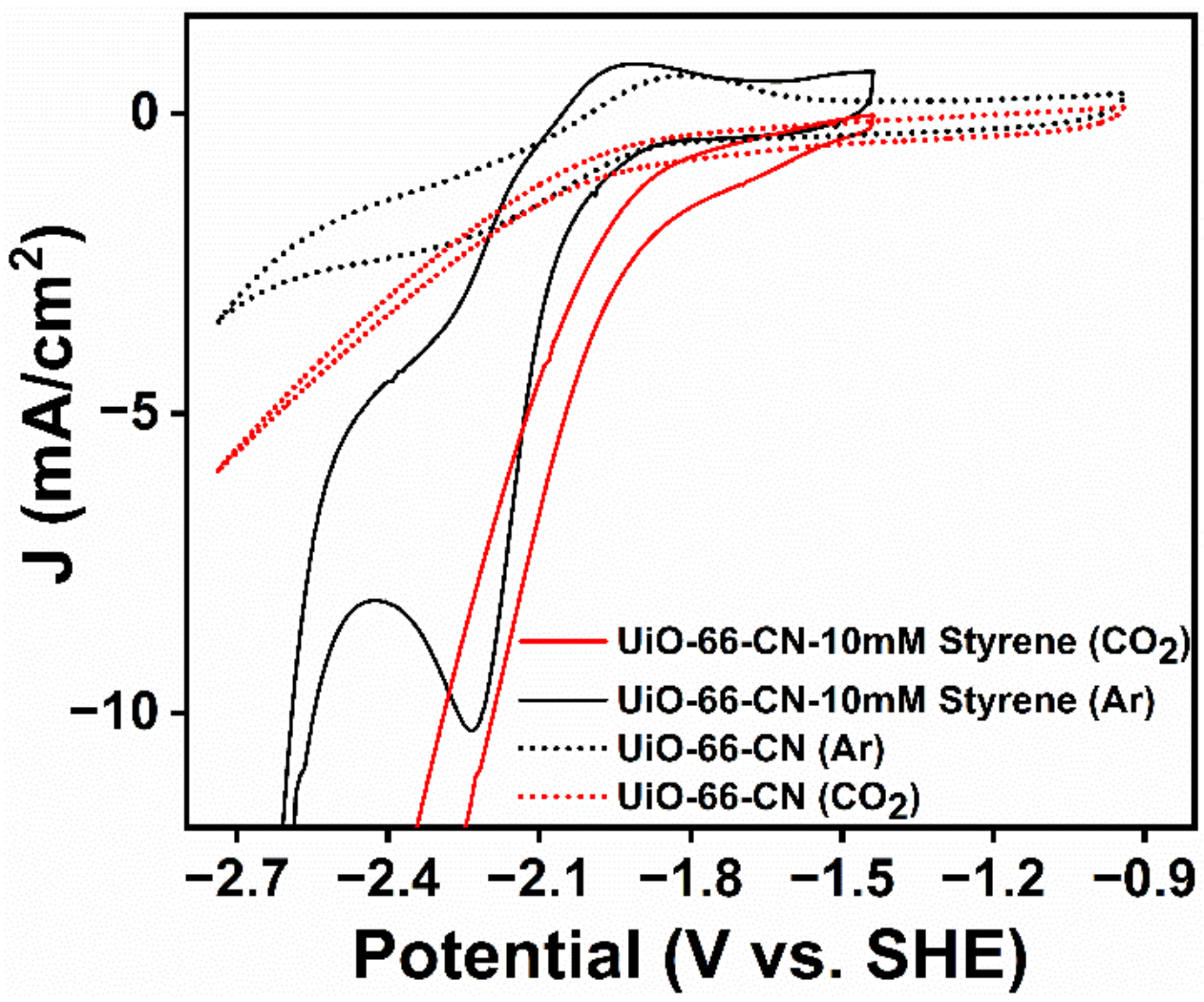

Figure S18. Cyclic voltammetry of UiO-66-CN electrode in presence of 10mM styrene, recorded at 500 mV s^{-1} in dry DMF under Ar (black) and CO_2 (red) environments.

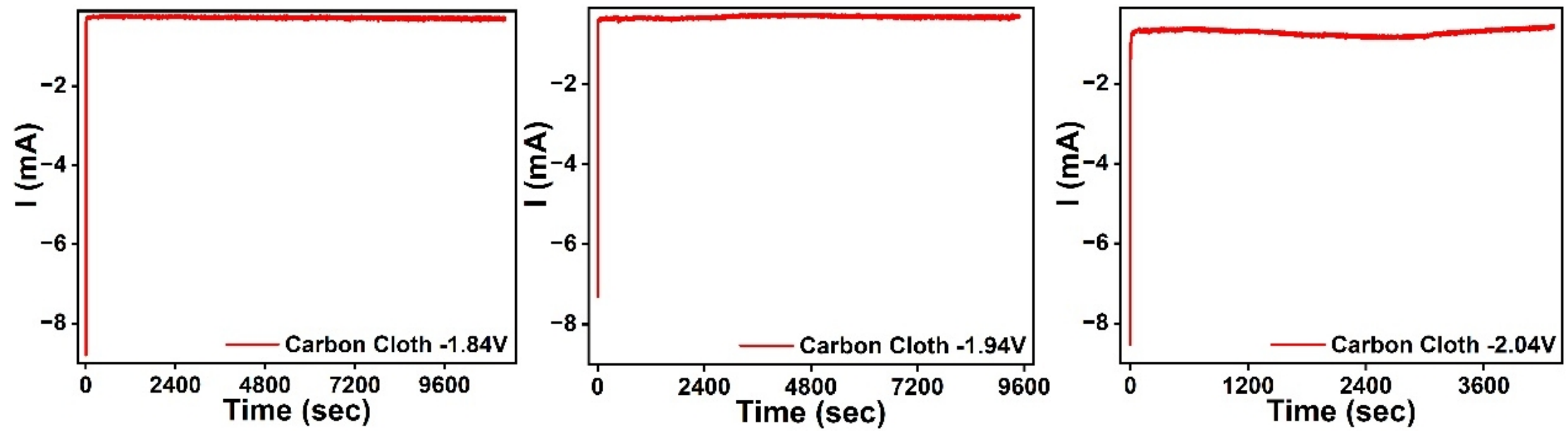

Figure S19. chronoamperometry measurements at various potentials (vs. SHE) containing 5mM styrene on carbon cloth (CC).

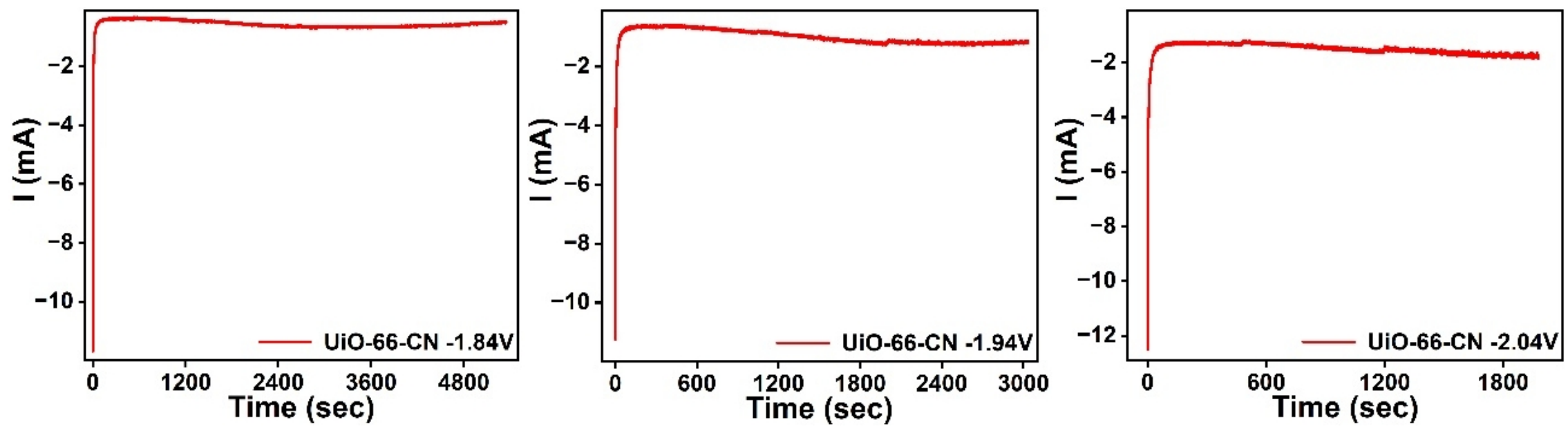

Figure S20. chronoamperometry measurements at various potentials (vs. SHE) containing 5mM styrene on CC-UiO-66-CN.

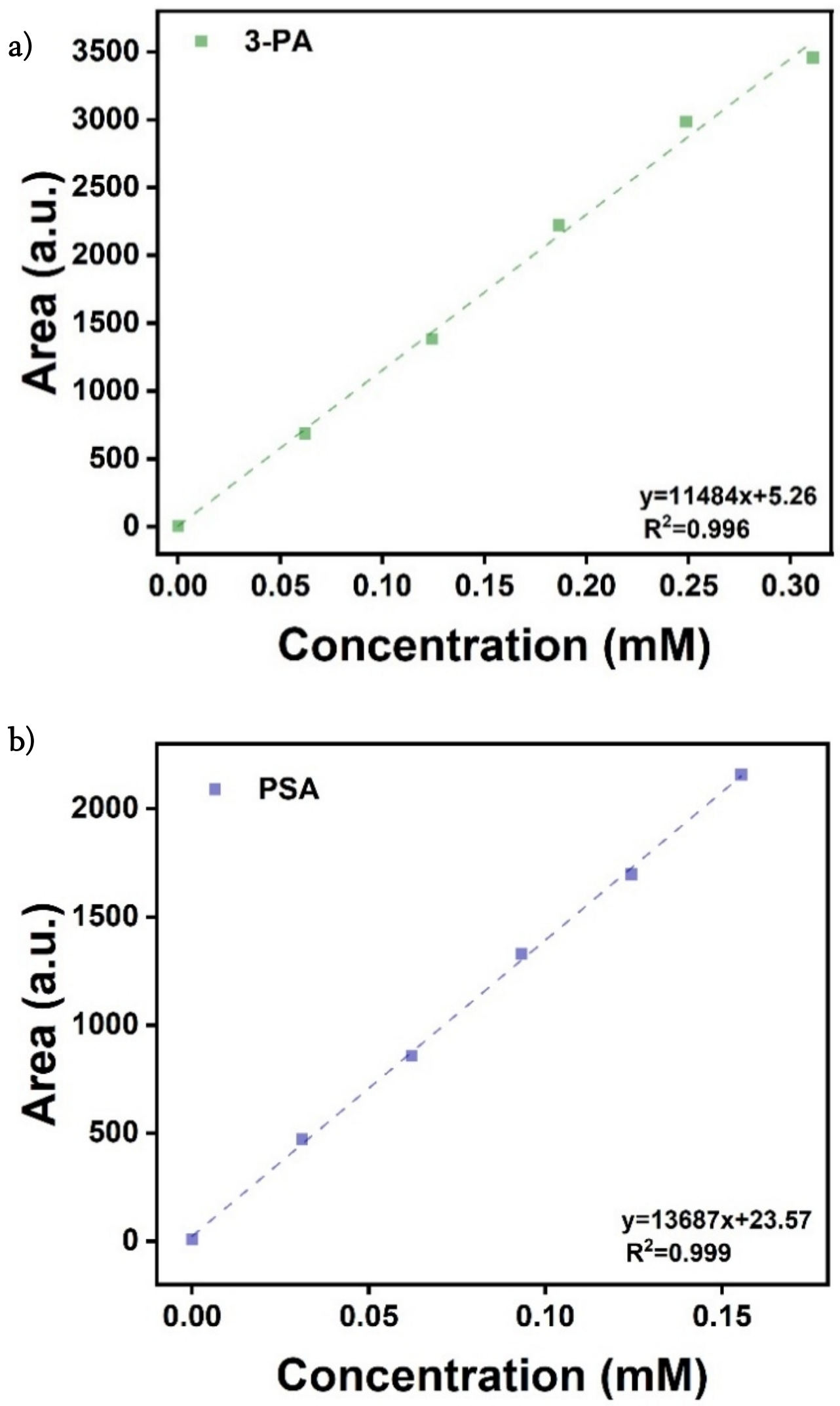

Figure S21. HPLC calibration plot for (a) 3-phenylpropanoic acid (3PA) and (b) phenylsuccinic acid (PSA).

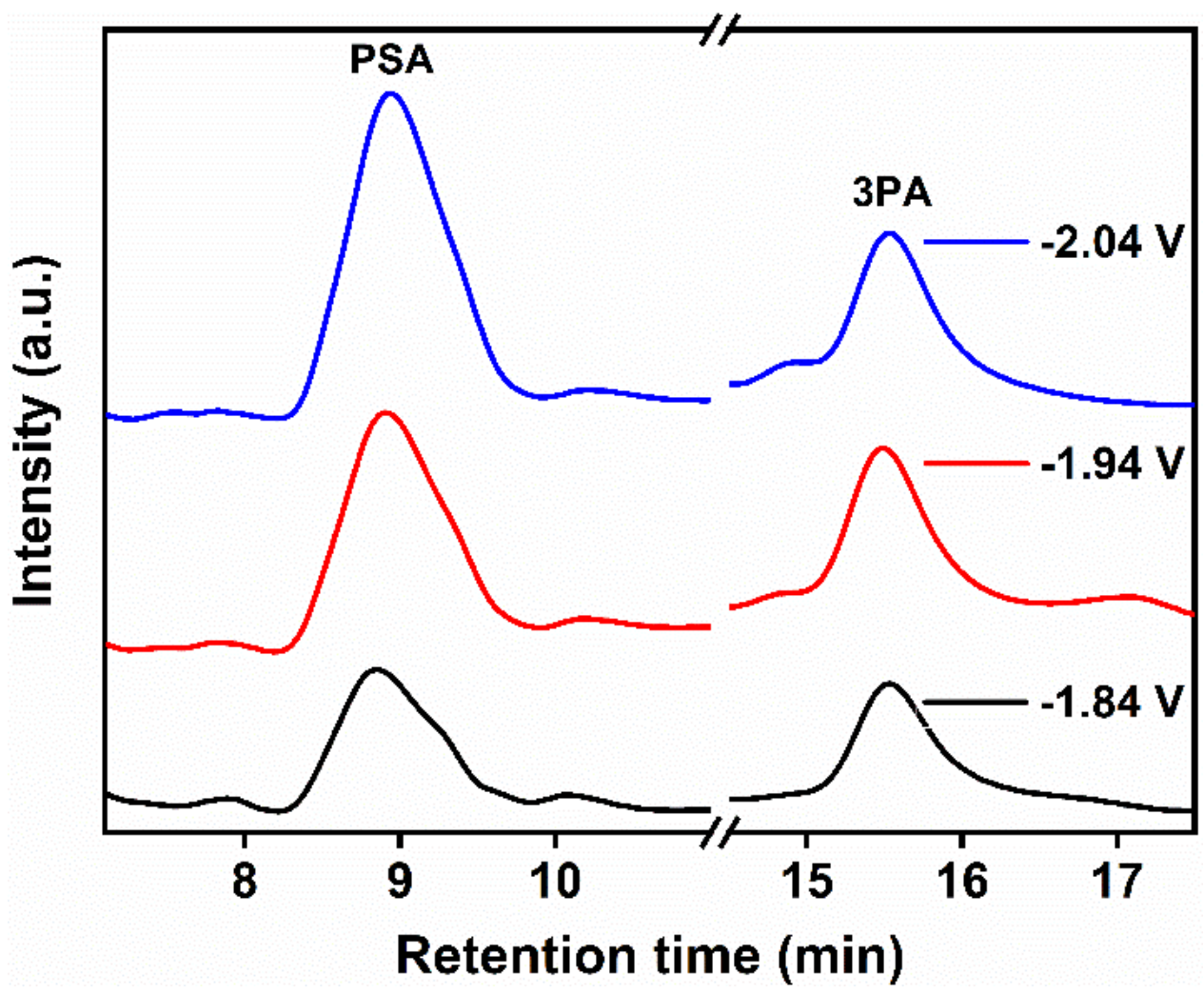

Figure S22. HPLC chromatogram of 3-phenylpropanoic acid (3PA) and phenylsuccinic acid (PSA) at different potential of UiO-66-CN electrodes.

Calculation of 3-phenylpropanoic acid (3PA) and phenylsuccinic acid (PSA) acid from HPLC:

For example, the area under the graph of PSA and 3PA at -2.04 V vs. SHE is: 1) **700,** 2) **339** respectively

Using the calibration curve equation and the area that was obtained from the HPLC we get the concentration of them in the solution.

1) ((700-23.57)/13687)*10 = **0.495mM**
2) ((339-5.26)/11484)*10 = **0.291mM**

(We multiply by 10 because we diluted the solution 10-fold with DI H_2O before injecting it into the HPLC).

Next, we divide the concentration by 1000 to convert it to M, and then divide by the total volume of the cell (10 mL).

1) (0.495mM/1000)*0.01L = **4.95E-4 mol**
2) (0.291mM/1000)*0.01L = **2.91E-4 mol**

1) The total electron of the reaction (2 e^- reaction, 3C charge) is **1.55E-5 mol**
2) The total electron of the reaction (1 e^- reaction, 3C charge) is **3.11E-5 mol**

1) %FE= (4.95E-4 mol /1.55E-5 mol)*100 ≈ **32%**
2) %FE= (2.91E-4 mol /3.11E-5 mol)*100 ≈ **9.4%**

Thus, the %FE of PSA is ≈32% and 3PA is ≈9.4%

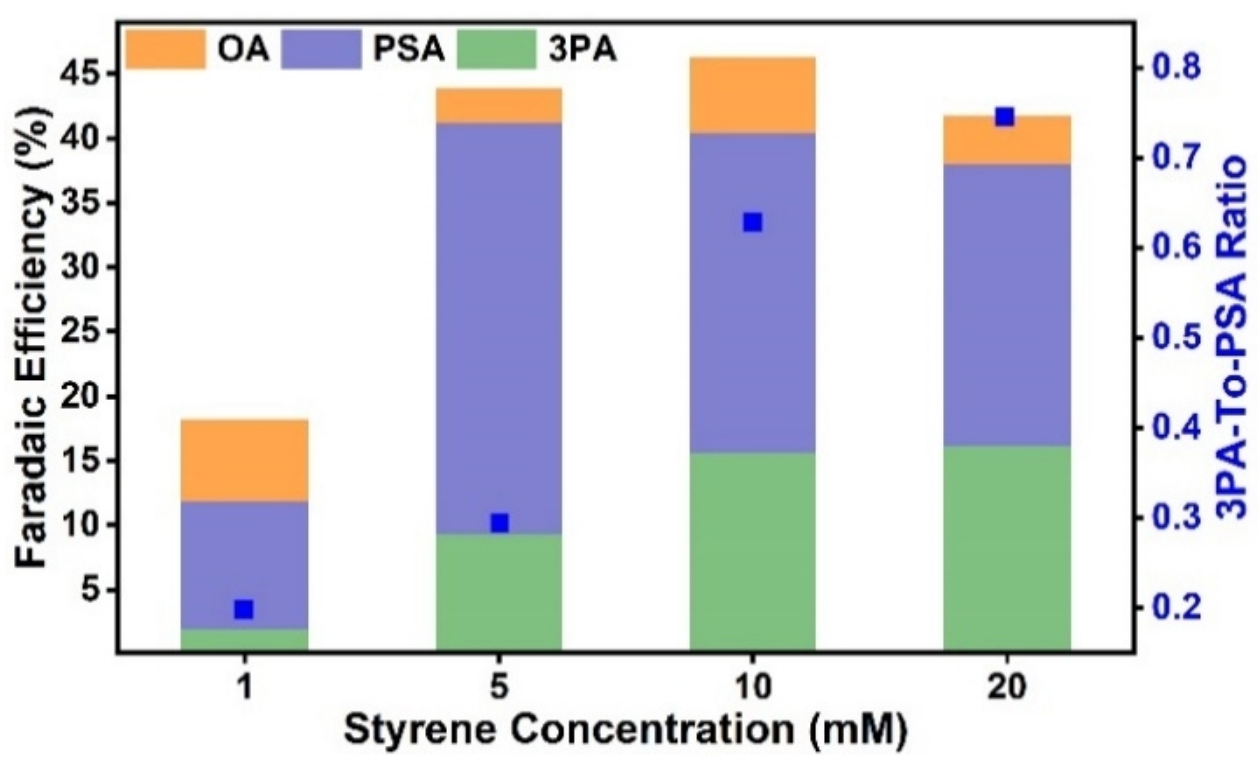

Figure S23. Effect of styrene concentration on product distribution and total Faradaic efficiency (applied potential of -2.04 V vs. SHE).

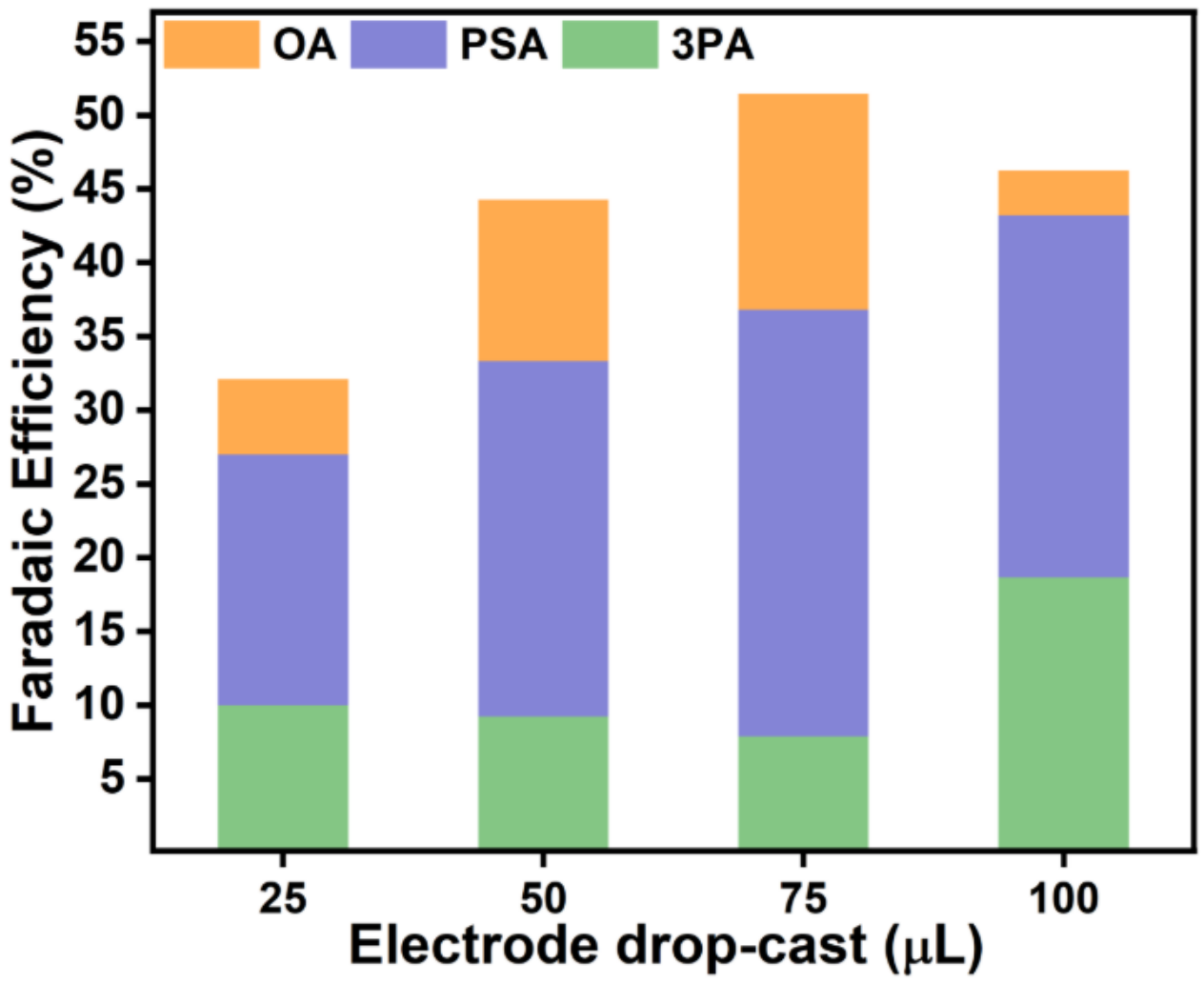

Figure S24. Total Faradaic efficiency of OA, PSA, and 3PA as a function of the UiO-66-CN drop-cast volume onto the electrode surface (applied potential of -2.04 V vs. SHE).

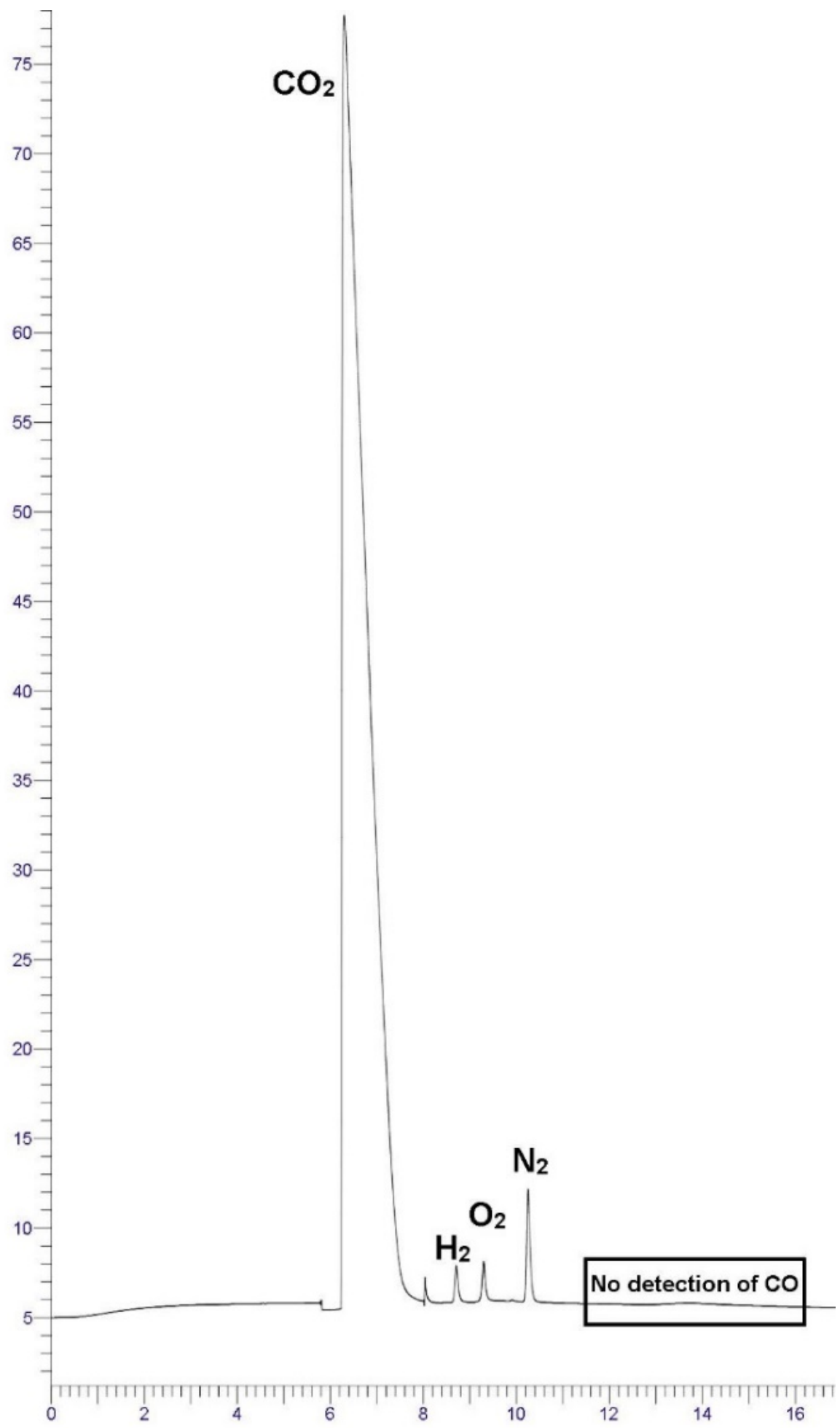

Figure S25. GC chromatogram of H_2 for Styrene experiment at -2.04 V vs. SHE using UiO-66-CN electrode.

Calculation of H_2 from GC:

For example, the area under the graph of H_2 at -2.04 V vs. SHE is: **9464**

Using the calibration curve equation and the area that was obtained from the GC we get the mol number of H_2 in the 0.5 mL syringe.

(9464/1.05E+12) = **9.013E-9 mol**

The total head space is 58 mL so we multiple by 116 to get the total mol number of H_2 in the cell.

9.013E-9 mol * (58 mL/0.5mL) = **1.046E-6 mol**

The total electron of the reaction (2 e^- reaction, 3C charge) is **1.55E-5 mol**

%FE= (1.046E-6 mol/1.55E-5 mol)*100 ≈ **7%**

Thus, the percentages of FE towed H_2 is 7%

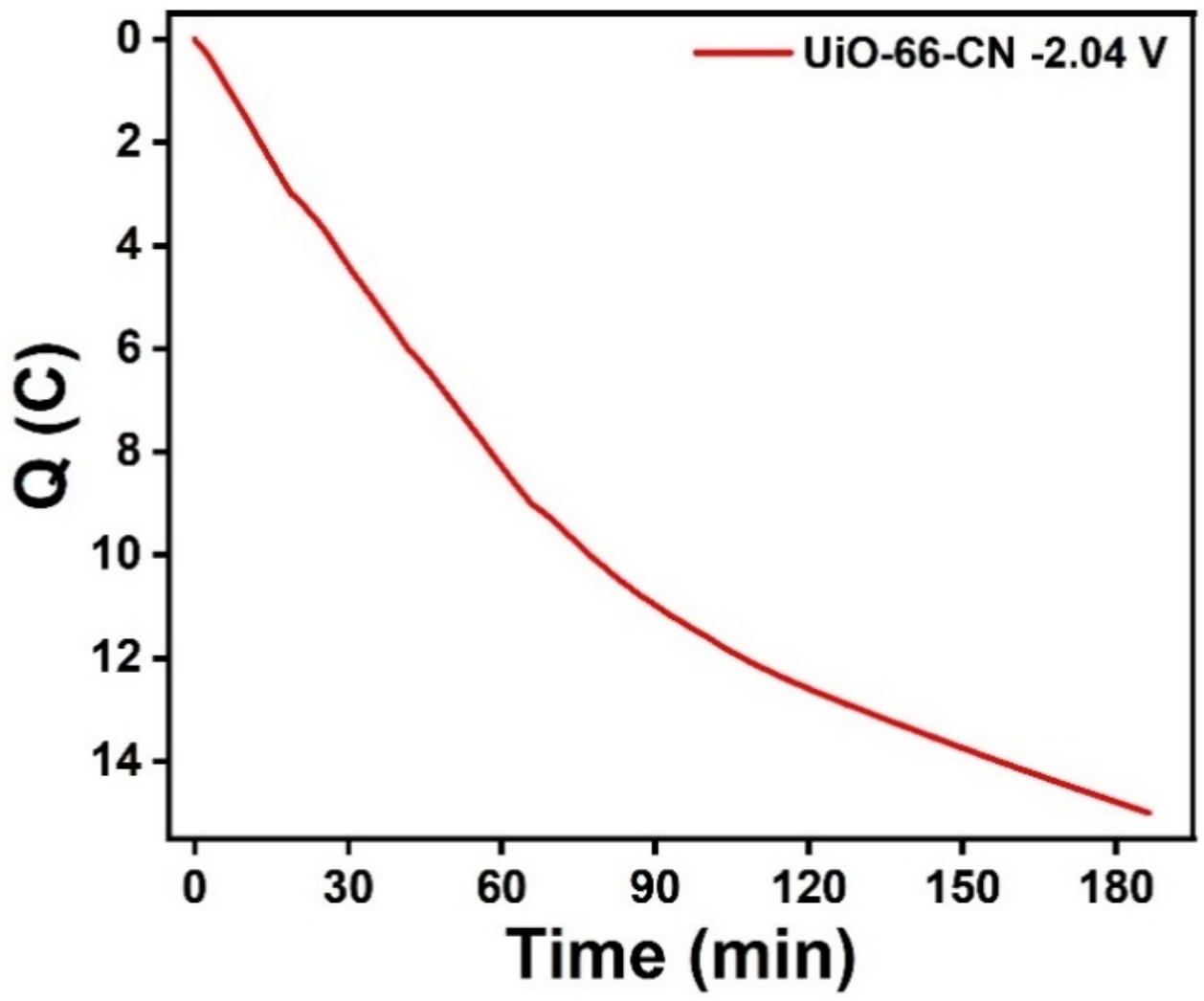

Figure S26. Chronoamperometry measurements at -2.04V (vs. SHE) containing 10mM styrene on CC-UiO-66-CN.

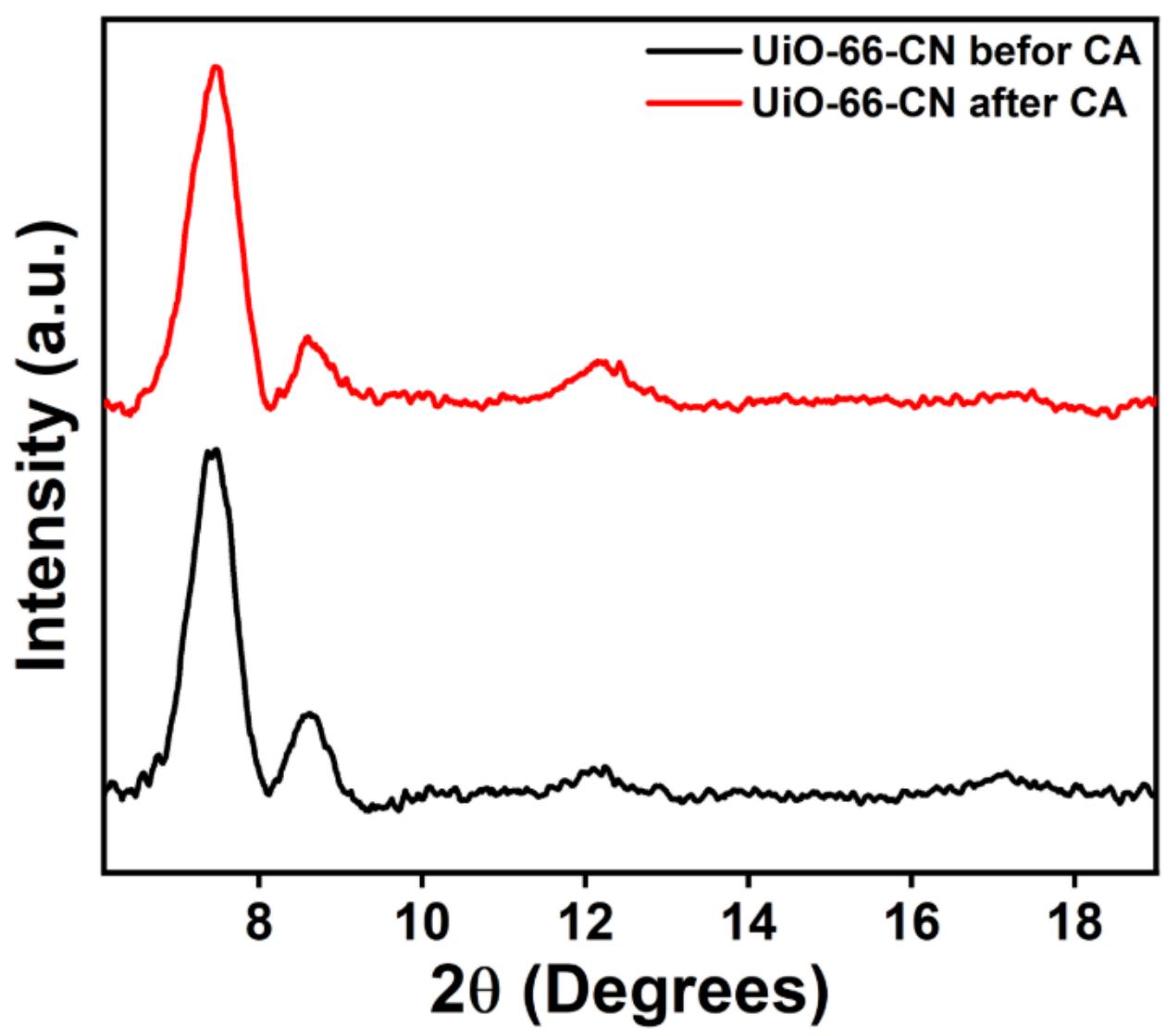

Figure S27. XRD patterns of UiO-66-CN before (black) and after (red) the chronoamperometric (CA) stability experiment.

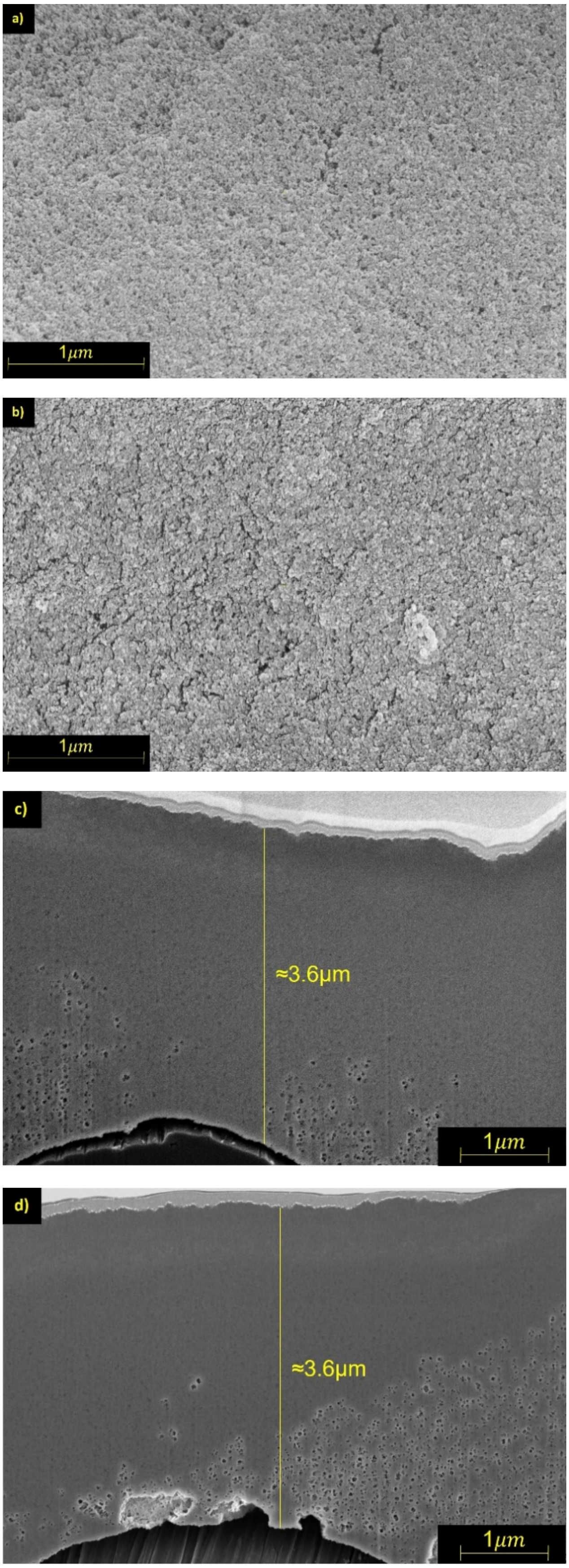
a)
1μm
b)
1μm
c)
≈3.6μm
1μm
d)
≈3.6μm
1μm

Figure S28. SEM-FIB cross-section images of UiO-66-CN on carbon cloth electrode before (a, c) and after (b, d) electrolysis.

Table S2. ICP-OES of UiO-66-CN after 3h of CA.

Sample	m [mg]	gr/L	V [L]	mol of Zr	mol of Zr in 1mg	mol of Zr_6	mol of BDC	BDC/Zr_6	4-CB/ Zr_6
UiO-66-CN	2.4	0.005256	0.0098	4.84 ∙ 10^{-6}	2.02 ∙ 10^{-6}	3.36 ∙ 10^{-7}	1.81 ∙ 10^{-6}	5.39	2.65
UiO-66-CN after 3h CA	1.8	0.015727	0.0098	1.45 ∙ 10^{-5}	8.05 ∙ 10^{-6}	1.34 ∙ 10^{-6}	7.02 ∙ 10^{-6}	5.24	2.35

Quantifying the loading of 4-cyanobenzoic acid (4-CB) groups in UiO-66-CN after 3h stability with the help of ICP-OES and ^{1}H-NMR.

As we did in a previous calculation (in table S1, and the calculation below S1), here too we will calculate the amount of 4-CB using the same method. The only different is that we took the electrodes after stability, We washed with Ethanol and dried thoroughly in oven at 60 °C , and place them into the solution. Each one weighed 0.6 mg. For ICP-OES we used 3 electrodes and for NMR we used only 1. In all of electrodes we drop cast 75 μL of UiO-66-CN in each side and did the same stability measurement.

Before electrochemistry measurement the loading level of 4-CB in UiO-66-CN was 2.65 per Zr_6-oxo node.

The number of 4-CB that underwent degradation is (2.65-2.35) = 0.3 per Zr_6-oxo node of UiO-66-CN.

Thus, the number of 4-CB per node that underwent degradation is 0.3 for UiO-66-CN gel after stability test.

The percentages of 4-CB that underwent degradation is ((0.3/2.65)*100%) = 11.3%.

Thus, the percentages of 4-CB that underwent degradation is ≈ 11% for UiO-66-CN gel after stability test.

References:

1. B. Bueken, N. Van Velthoven, T. Willhammar, T. Stassin, I. Stassen, D. A. Keen, G. V. Baron, J. F. M. Denayer, R. Ameloot, S. Bals, D. De Vos and T. D. Bennett, *Chemical Science*, 2017, **8**, 3939-3948.
2. S. Mukhopadhyay, M. S. Naeem, G. Shiva Shanker, A. Ghatak, A. R. Kottaichamy, R. Shimoni, L. Avram, I. Liberman, R. Balilty, R. Ifraemov, I. Rozenberg, M. Shalom, N. López and I. Hod, *Nature Communications*, 2024, **15**, 3397.
3. R. Balilty, I. Liberman, R. Shimoni and I. Hod, *Langmuir*, 2025, **41**, 34522-34532.
4. V. Dhayalan, S. C. Gadekar, Z. Alassad and A. Milo, *Nature Chemistry*, 2019, **11**, 543-551.
5. G. R. Buettner, *Free Radical Biology and Medicine*, 1987, **3**, 259-303.
6. L. Li and C. Cui, 2025, **64**, e202505042.
7. D. W. H. Merkx, L. Plankensteiner, Y. Yu, P. A. Wierenga, M. Hennebelle and J. P. M. Van Duynhoven, *Food Chemistry*, 2021, **334**, 127578.
8. L. Pichereau, L. Fillaud, N. Kostopoulos, E. Maisonhaute, T. Cauchy, M. Allain, J.-M. Noël, C. Gautier and T. Breton, *The Journal of Physical Chemistry Letters*, 2022, **13**, 11866-11871.

9. E. Chernikova, V. Golubev, A. Filippov, C. Y. Lin and M. L. Coote, *Polymer Chemistry*, 2010, **1**, 1437-1440.

10. M. L. Pegis, J. A. S. Roberts, D. J. Wasylenko, E. A. Mader, A. M. Appel and J. M. Mayer, *Inorganic Chemistry*, 2015, **54**, 11883-11888.
